# Amino Alcohols from the Ascidian *Pseudodistoma* sp

**DOI:** 10.3390/md12063754

**Published:** 2014-06-24

**Authors:** Tae Hyung Won, Minjung You, So-Hyoung Lee, Boon Jo Rho, Dong-Chan Oh, Ki-Bong Oh, Jongheon Shin

**Affiliations:** 1 Natural Products Research Institute, College of Pharmacy, Seoul National University, San 56-1, Sillim, Gwanak, Seoul 151-742, Korea; E-Mails: wth123@snu.ac.kr (T.H.W.); lovemin90@snu.ac.kr (M.Y.); dongchanoh@snu.ac.kr (D.-C.O.); 2 Department of Agricultural Biotechnology, College of Agriculture and Life Science, Seoul National University, San 56-1, Sillim, Gwanak, Seoul 151-921, Korea; E-Mail: awhee84@naver.com; 3 Department of Biological Science, College of Life Science, Ewha Womans University, 52, Ewhayeodae-gil, Seodaemun, Seoul 120-750, Korea; E-Mail: nhm@ewha.ac.kr

**Keywords:** amino alcohols, pseudoaminols, *Pseudodistoma* sp., stereochemistry, bioactivities

## Abstract

Seven new amino alcohol compounds, pseudoaminols A–G (**1**–**7**), were isolated from the ascidian *Pseudodistoma* sp. collected off the coast of Chuja-do, Korea. Structures of these new compounds were determined by analysis of the spectroscopic data and from chemical conversion. The presence of an *N*-carboxymethyl group in two of the new compounds (**6** and **7**) is unprecedented among amino alcohols. Several of these compounds exhibited moderate antimicrobial activity and cytotoxicity, as well as weak inhibitory activity toward Na^+^/K^+^-ATPase.

## 1. Introduction

Linear amino alcohols possessing a hydroxy and an amino group are widely distributed in marine organisms [1]. Since the first isolation of amino alcohols from *Xestospongia* sp. [2], several related compounds have been reported from marine ascidians [3,4,5,6,7,8,9,10], sponges [11,12,13,14,15], clams [16], and microorganisms [17]. These compounds exhibited interesting antimicrobial and cytotoxic activities. These metabolites are structurally related sphingosine derivatives, which have long been known as central structural elements of sphingolipids and as important cell membrane components that function in cell recognition and cell regulation [18].

**Figure 1:**
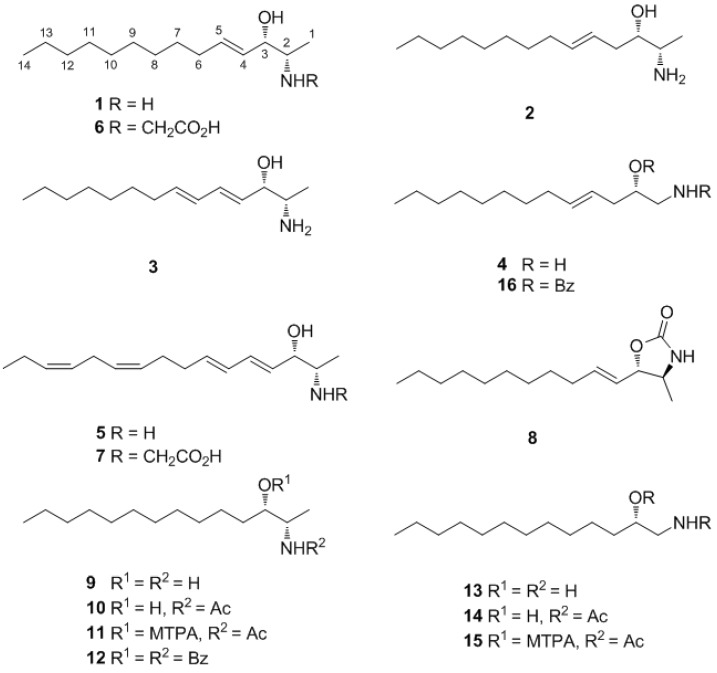
Structures of compounds **1**–**16**.

During the course of searching for bioactive metabolites from Korean marine invertebrates, we encountered the reddish-orange ascidian *Pseudodistoma* sp. off the coast of Chuja-do, Korea, and its organic extract exhibited significant cytotoxicity (LD_50_ 14.6 μg/mL) against the A549 cell line. Bioassay-guided separation of the crude extract using various chromatographic techniques yielded several nitrogenous lipids. We report here the isolation and structural elucidation of seven new amino alcohols, pseudoaminols A–G (**1**–**7**) (Figure 1). To the best of our knowledge, the *N*-carboxymethyl functionality of pseudoaminols F (**6**) and G (**7**) possibly derived from the amino acid glycine, has not been previously reported among amino alcohols. Several of these compounds exhibited moderate cytotoxicity and inhibition against diverse bacterial strains and the enzyme Na^+^/K^+^-ATPase.

## 2. Results and Discussion

The ascidian specimens were lyophilized, macerated, and repeatedly extracted with MeOH and CH_2_Cl_2_. The combined extracts were separated by solvent partitioning, followed by reversed-phase flash chromatography. The polar fractions with amino alcohol mixtures, as determined by NMR analysis, were separated via reversed-phase HPLC to yield seven compounds.

Pseudoaminol A (**1**) was isolated as an amorphous solid with a molecular formula of C_14_H_29_NO, as determined by HRFABMS analysis. The ^13^C NMR spectrum of this compound showed signals corresponding to two olefinic (δ_C_ 137.2 and 129.8), two methine (δ_C_ 75.0 and 53.3), and ten upfield carbons (δ_C_ 33.3–14.4) (Table 1). The linear lipid nature of this compound was apparent from its lack of quaternary carbons and the presence of an oxygen and a nitrogen at the methine carbons at δ_C_ 75.0 and 53.3, respectively, as deduced from their chemical shifts. The sequentially correlated proton signals at δ_H_ 5.83, 5.42, 3.88, 3.08, and 1.22 and the corresponding carbon signals at δ_C_ 137.2, 129.8, 75.0, 53.3, and 15.7, respectively, were attributed to a 2-amino-3-hydroxy-alkyl moiety directly linked to a disubstituted double bond from the COSY, HSQC, and HMBC data (Table 2). The combined 2-D NMR data also revealed that the remaining upfield protons were linearly assembled to form an eight-carbon alkyl chain attached at the previously determined double bond. The *E* configuration was assigned to this double bond based on the large vicinal coupling constants between the olefinic protons (*J*_4,5_ = 15.5 Hz), as well as the NOESY cross-peaks at H-3/H-5 and H-4/H-6. In conclusion, the planar structure of **1** was determined to be (*E*)-2-aminotetradec-4-en-3-ol.

**Table 1:** ^13^C NMR (ppm, mult) Assignments for Compounds **1**–**7** *^a^*.

Position	1	2	3	4	5	6	7
1	15.7, CH_3_	15.8, CH_3_	15.7, CH_3_	45.3, CH_2_	15.7, CH_3_	12.7, CH_3_	12.9, CH_3_
2	53.3, CH	52.7, CH	53.3, CH	68.7, CH	53.6, CH	57.8, CH	57.8, CH
3	75.0, CH	73.1, CH	74.6, CH	39.4, CH_2_	74.6, CH	72.2, CH	72.1, CH
4	129.8, CH	38.0, CH_2_	129.7, CH	125.6, CH	130.1, CH	129.4, CH	130.4, CH
5	137.2, CH	125.7, CH	135.6, CH	135.4, CH	135.5, CH	134.0, CH	132.8, CH
6	33.3, CH_2_	135.5, CH	130.6, CH	33.6, CH_2_	130.9, CH	31.2, CH_2_	129.8, CH
7	30.1 *^b^*, CH_2_	33.7, CH_2_	137.8, CH	30.5 *^b^*, CH_2_	137.0, CH	28.5 *^b^*, CH_2_	134.8, CH
8	30.3 *^b^*, CH_2_	30.6 *^b^*, CH_2_	33.6, CH_2_	30.4 *^b^*, CH_2_	33.7, CH_2_	28.5 *^b^*, CH_2_	32.1, CH_2_
9	30.4 *^b^*, CH_2_	30.6 *^b^*, CH_2_	30.3 *^b^*, CH_2_	30.3 *^b^*, CH_2_	27.9, CH_2_	28.6 *^b^*, CH_2_	26.4, CH_2_
10	30.6 *^b^*, CH_2_	30.4 *^b^*, CH_2_	30.3 *^b^*, CH_2_	30.2 *^b^*, CH_2_	129.9, CH	28.8 *^b^*, CH_2_	129.0, CH
11	30.7 *^b^*, CH_2_	30.3 *^b^*, CH_2_	30.3 *^b^*, CH_2_	32.9, CH_2_	129.7, CH	28.9 *^b^*, CH_2_	128.3, CH
12	33.1, CH_2_	33.1, CH_2_	33.0, CH_2_	23.6, CH_2_	26.4, CH_2_	31.6, CH_2_	25.2, CH_2_
13	23.7, CH_2_	23.7, CH_2_	23.7, CH_2_	14.3, CH_3_	128.3, CH	22.1, CH_2_	127.1, CH
14	14.4, CH_2_	14.4, CH_3_	14.4, CH_3_		132.6, CH	13.9, CH_3_	131.5, CH
15					21.5, CH_2_		20.1, CH_2_
16					14.6, CH_3_		14.2, CH_3_
1′						46.5, CH_2_	46.7, CH_2_
2′						168.0, C	168.5, C

*^a^* Data were obtained in MeOH-*d*_4_ (**1**–**5**) and DMSO-*d*_6_ (**6**, **7**) solutions; *^b^* Interchangeable signals.

**Scheme 1:**
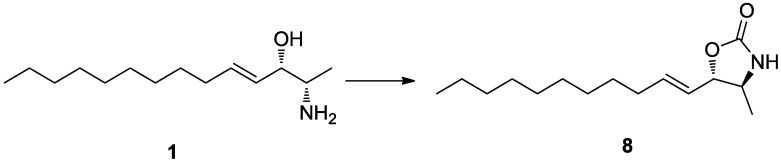
*Reagents and Conditions*: 1,1′-carbonyldiimidazole, CH_2_Cl_2_, rt, 2 h.

Pseudoaminol A (**1**) possessed asymmetric carbon centers at C-2 and C-3. The relative configurations were determined by vicinal proton-proton coupling constants and NOESY experiments performed on the corresponding oxazolidinone derivative **8** of **1**, which was prepared by treatment with 1,1′-carbonyl diimidazole (Scheme 1). Irradiation of the H-1 and H-4 signals at δ_H_ 1.22 and 5.42, respectively, showed a coupling constant of *J*_2,3_ = 7.5 Hz, which indicated a *threo* configuration for C-2 and C-3. The alternative *erythro* configuration would have given a *J* of 4.5 Hz [19]. This interpretation was also confirmed by the cross-peaks at H-1/H-3 and H-2/H-5 in the NOESY data.

**Table 2:** ^1^H NMR (ppm, mult) Assignments for Compounds **1**–**3** *^a^*.

Position	1	2	3
1	1.22, d (7.0)	1.26, d (7.0)	1.22, d (7.0)
2	3.08, dq (7.5, 7.0)	3.11, dq (3.0, 7.0)	3.10, dq (7.5, 7.0)
3	3.88, dd (7.0, 7.0)	3.50, dd (5.0, 7.0, 7.0)	3.90, dd (7.5, 7.5)
4	5.42, dd (7.5, 15.5)	2.33, ddd (7.0, 7.0, 14.5); 2.17, ddd (7.0, 7.0, 14.5)	5.51, dd (7.5, 15.0)
5	5.83, td (7.0, 15.5)	5.48, dt (7.0, 7.0, 15.0)	6.33, dd (11.0, 15.0)
6	2.09, td (7.5, 7.0)	5.57, dt (7.0, 7.0, 15.0)	6.00, dd (11.0, 15.0)
7	1.34, tt (7.5, 7.5)	2.03, dt (7.0, 7.0)	5.78, dd (7.5, 15.0)
8	1.23–1.27, m	1.26–1.32, m	2.09, dt (7.0, 7.0)
9	1.23–1.27, m	1.26–1.32, m	1.38, tt (7.0, 7.0)
10	1.23–1.27, m	1.26–1.32, m	1.26–1.32, m
11	1.23–1.27, m	1.26–1.32, m	1.26–1.32, m
12	1.23–1.27, m	1.26–1.32, m	1.26–1.32, m
13	1.23–1.27, m	1.26–1.32, m	1.26–1.32, m
14	0.89, t (7.0)	0.89, t (7.0)	0.89, t (7.0)

*^a^* Data were obtained in MeOH-*d*_4_ solutions.

**Scheme 2:**
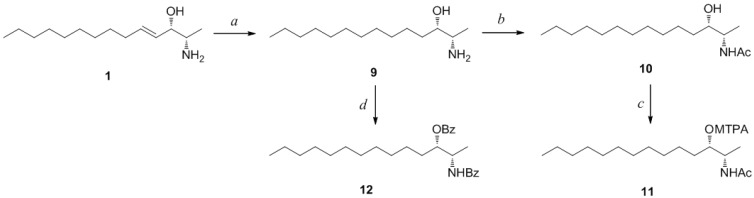
*Reagents and Conditions*: (a) H_2_, Pd/C, MeOH, rt, 2 h; (b) acetic anhydride, pyridine, rt, 1 h; (c) MTPA-Cl, pyridine, DMAP, rt, 1 h; (d) benzoyl chloride, pyridine, DMAP, rt, 3 h.

**Figure 2:**
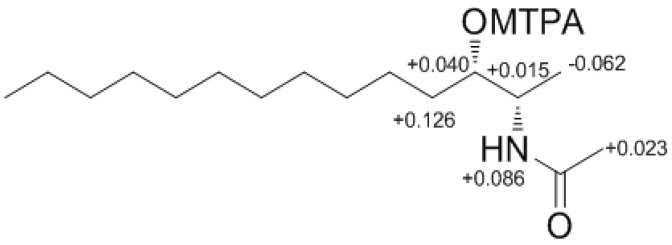
The Result (∆δ**_11S−11R_**) of MTPA esterification for compound **11**.

Determination of the absolute configuration of **1** was initially attempted by application of the Mosher method, which involved MTPA-Cl esterification of the C-3 hydroxyl group. To avoid possible steric hindrance by the neighboring C-4 double bond and concomitant MTPA esterification at the C-2 amine group, **1** was sequentially hydrogenated (**9**) and acetylated (**10**) prior to MTPA esterification (Scheme 2). However, the MTPA esters of compound **10** exhibited irregular diamagnetic proton shifts, possibly due to the spatial crowding between C-2 and C-3 (Figure 2). These results contradict previous determinations of absolute configurations using the Mosher method for similar amino alcohols [6,10,14].

**Figure 3:**
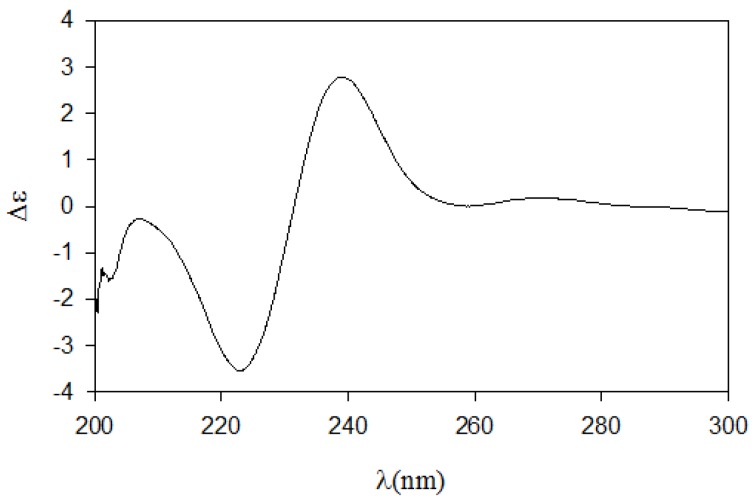
The CD spectrum of compound **12**.

Next, we employed the recently proposed CD method, which has been used to unambiguously assign both relative and absolute configurations of diastereomeric *N*,*O*-dibenzoyl-2-amino-3-alkanols [7]. The CD spectrum of the dibenzoyl derivative **11** obtained by treatment of **8** with benzoyl chloride (Scheme 2) closely matched the (2*S*,3*S*)-*threo* model because it exhibited the same sign for the negative and positive cotton effects at approximately 220 nm and 240 nm, respectively, and because it exhibited the same band magnitude (Figure 3). Thus, the absolute configurations of pseudoaminol A (**1**) were determined to be 2*S* and 3*S.*

The molecular formula of pseudoaminol B (**2**) was deduced as C_14_H_29_NO by HRFABMS analysis, which was the same as that of **1**. The NMR data of this compound were also similar to those of **1**; the shifts of a methylene resonance (δ_C_ 38.0 and δ_H_ 2.17 and 2.33) were the most noticeable differences between the spectra of **1** and **2** (Table 1 and Table 2). On the basis of the results of combined 2-D NMR experiments, pseudoaminol B (**2**) was determined to be a derivative of **1**, with migration of the C-4 double bond to C-5. The *E* configuration was assigned to this double bond on the basis of the large proton coupling constants (*J*_4,5_ = 15.5 Hz).

Despite their structural similarity, the specific rotation of **2** was opposite that of **1 (**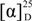 −4.4 and +5.1 for **1** and **2**, respectively), which led us to conduct a detailed investigation. Thus, **2** was hydrogenated and converted to the dibenzoyl derivative, as shown in Scheme 2. After each step, the specific rotations for the corresponding derivatives of **1** and **2** were compared. The specific rotations of the hydrogenated derivatives (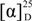 −6.9 and −7.1, respectively) and dibenzoyl analogs (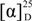 −12.3 and −12.4, respectively) were virtually identical, regardless of their origin. The CD spectra of the dibenzoyl derivatives of **1** and **2** were also identical (See Supplementary Information, Figure S1). In addition to similar ^13^C and ^1^H NMR chemical shifts, chemical derivatization allowed unambiguous assignment of the same absolute configuration to **1** and **2**. The significant difference in the specific optical rotation between these compounds is a result of the direct influence of the neighboring electron-rich double bond.

Pseudoaminol C (**3**) exhibited a molecular formula of C_14_H_27_NO, as determined by HRFABMS analysis, indicating that it possessed two fewer protons than **1**. The NMR data for this compound showed four olefinic signals (δ_C_ 137.8, 135.6, 130.6, and 129.7; δ_H_ 6.33, 6.00, 5.78, and 5.51), which were assigned to a conjugated diene moiety on the basis of the COSY and HSQC analyses. Aided by the HMBC data, the structure of **3** was determined to be a linear amino alcohol derivative with a conjugated diene at C-4. The configurations of both double bonds were assigned as *E* on the basis of the large coupling constants between the olefinic protons (*J*_4,5_ = *J*_6,7_ = 15.0 Hz).

The molecular formula of pseudoaminol E (**4**) was determined to be C_13_H_27_NO by HRFABMS analysis. The most conspicuous difference between the NMR results for **4** and **2** was the absence of the C-1 terminal methyl signal (δ_C_ 15.8 and δ_H_ 1.26), which coincided with the loss of a carbon on the basis of the MS analysis. In addition, signals of nitrogenous terminal methylene protons (δ_H_ 2.74 and 3.00) were present in the ^1^H NMR spectrum (Table 3). Based on the combined 2-D NMR analyses, the structure of **4** was determined to be a linear C_13_ amino alcohol derivative with a terminal amino group. The geometry of the double bond was assigned as *E* on the basis of the proton coupling constants (*J*_4,5_ = 15.0 Hz).

**Table 3:** ^1^H NMR (ppm, mult) Assignments for Compounds **4**–**7** *^a^*.

Position	4	5	6	7
1	2.74, dd (10.0, 13.0); 3.00, dd (3.0, 13.0)	1.23, d (7.0)	1.10, d (7.0)	1.22, d (7.0)
2	3.76, dddd (3.0, 7.0, 7.0, 10.0)	3.11, dq (7.5, 7.0)	2.98, dq (7.5, 7.0)	2.99, dq (7.5, 7.0)
3	2.24, ddd (7.0, 7.0, 14.0); 2.19, ddd (7.0, 7.0, 14.0)	3.96, dd (7.5, 7.5)	3.88, dd (7.5, 7.5)	4.02, dd (7.5, 7.5)
4	5.44, ddd (7.0, 7.0, 15.0)	5.53, dd (7.5, 15.0)	5.37, dd (15.0, 7.5)	5.53, dd (15.0, 7.5)
5	5.56, dt (15.0, 7.0)	6.32, dd (10.5, 15.0)	5.68, ddd (7.0, 7.0, 15.0)	6.21, dd (11.0, 15.0)
6	2.02, dt (7.0, 7.0)	6.11, dd (10.5, 15.0)	2.00, ddd (7.0, 7.0, 15.0)	6.06, dd (11.0, 15.0)
7	1.35, tt (7.0, 7.0)	5.78, dt (15.0, 7.0)	1.33, m	5.73, dd (7.5, 7.5)
8	1.25–1.33, m	2.16, s	1.23–1.28, m	2.11, br s
9	1.25–1.33, m	2.16, s	1.23–1.28, m	2.11, br s
10	1.25–1.33, m	5.35, m	1.23–1.28, m	5.33, m
11	1.25–1.33, m	5.35, m	1.23–1.28, m	5.33, m
12	1.25–1.33, m	2.77, dd (6.0, 6.0)	1.23–1.28, m	2.73, dd (6.0, 6.0)
13	0.89, t (7.0)	5.31, m	1.23–1.28, m	5.25, dt (10.0, 6.0)
14		5.35, m	0.84, t (7.0)	5.33, m
15		2.06, dq (7.0, 7.0)		2.02, dq (7.0, 7.0)
16		0.96, t (7.0)		0.91, t (7.0)
1′			3.35, d (15.0)	3.34, d (15.0)
			3.23, d (15.0)	3.23, d (15.0)

*^a^* Data were obtained in MeOH-*d*_4_ (**4**, **5**) and DMSO-*d*_6_ (**6**, **7**) solutions.

**Scheme 3:**
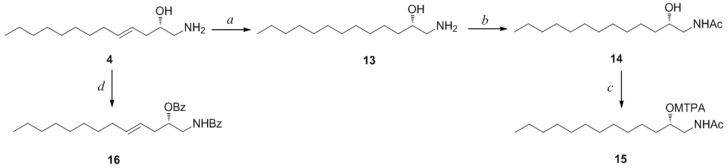
*Reagents and Conditions*: (a) H_2_, Pd/C, MeOH, rt, 2 h; (b) acetic anhydride, pyridine, rt, 1 h; (c) MTPA-Cl, pyridine, DMAP, rt, 1 h; (d) benzoyl chloride, pyridine, DMAP, rt, 3 h.

**Figure 4:**
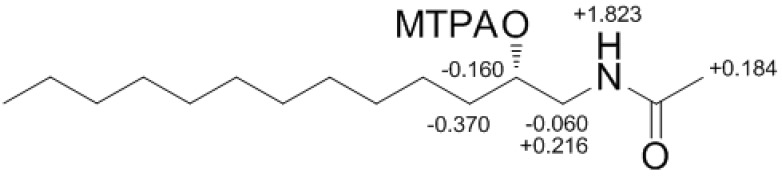
The Result (∆δ**_15S-15R_**) of MTPA esterification for compound **15**.

Compound **4** contained an asymmetric carbon center at the C-2 oxymethine. The Mosher method was used to determine the absolute configuration of this carbon. Similar to the case of **1**, compound **4** was sequentially hydrogenated (**13**) and acetylated (**14**) prior to MTPA esterification in order to avoid potential steric hindrance from the neighboring C-4 double bond and additional MTPA esterification at the C-1 amine group (Scheme 3). The ∆δ values of the signals of the protons near the hydroxyl group at C-2 were negative to the left and positive to the right, suggesting the 2*S* configuration, with the exception of one of the H-1 methylene protons (Figure 4). The absolute configuration was confirmed by CD method on a synthetic derivative. The CD spectrum of the dibenzoyl derivative (**16**) obtained by treatment of **4** with benzoyl chloride (Scheme 3) showed a positive cotton effect (222 nm (∆ε −5.8) and 239 nm (∆ε +7.1)), which was opposite to the previously reported 2*R* model [20] (See Supplementary Information, Figure S2). Overall, the absolute configuration was determined to be *S* in accord with the configurations of other amino alcohols at the oxymethine position.

The molecular formula of pseudoaminol E (**5**) was deduced as C_16_H_28_NO by HRFABMS analysis. The ^13^C NMR spectrum of this compound showed signals corresponding to eight olefinic (δ_C_ 137.0–128.3), two methine (δ_C_ 53.6 and 74.6), and six upfield carbons (δ_C_ 33.7–14.6). The presence of eight methylene signals at δ_H_ 2.77–2.06 in the ^1^H NMR spectrum indicated the presence of allylic and *bis*-allylic methylenes and provided insight into the double-bond system. On the basis of the combined results of 2-D NMR analyses, the conjugated double bond was located at C-4 and the isolated double bonds were located at C-10 and C-13. Overall, the structure of compound **5** was determined to be a highly unsaturated, linear amino alcohol possessing a conjugated diene moiety. The configurations of the four double bonds were assigned on the basis of the proton coupling constants and carbon chemical shifts. *E* configuration was assigned to the C-4 and C-6 double bonds based on the large vicinal proton coupling constants (*J*_4,5_ = *J*_6,7_ = 15.0 Hz). In contrast, the *Z* configuration of the C-10 and C-13 double bonds was determined on the basis of the upfield chemical shifts of the allylic and *bis*-allylic carbons (δ_C_ 27.9, 26.4, and 21.5 for C-9, C-12, and C-15, respectively) in the ^13^C NMR spectrum [21]. The severely overlapping olefinic proton signals hindered both the accurate measurement of the proton coupling constants and NOESY analysis.

In addition to the linear amino alcohols, structurally related metabolites with unusual functionalities were isolated and analyzed. The molecular formula of pseudoaminol F (**6**) was established as C_16_H_31_NO_3_ by HRFABMS analysis. The ^13^C NMR spectrum of this compound was very similar to that of **1**; the presence of a carbonyl and a methylene carbon at δ_C_ 168.0 and 46.5, respectively, were the most noticeable differences. A difference was also observed in the ^1^H NMR spectrum, as proton signals of additional methylenes were observed at δ_H_ 3.35 (1H, d, *J* = 15.0 Hz) and 3.23 (1H, d, *J* = 15.0 Hz) (Table 3). A strong absorption band was also present at 1747 cm^−1^ in the IR spectrum; this result, in conjunction with the MS results, indicated that the carbonyl group was a carboxylic acid. The combined 2-D NMR experiments of **6** revealed the presence of the same linear carbon framework as **1**. The new functional group was observed to be a carboxymethyl group directly attached to the 2-amino group on the basis of the long-range carbon-proton correlations at H-2/C-1′, H-1′/C-2, and H-1′/C-2′ in the HMBC spectra. To the best of our knowledge, this *N*-carboxymethyl group possibly derived from amino acid glycine, is the first example of its type among amino alcohols.

Related compound **7** was determined to have a molecular formula of C_18_H_29_NO_3_ by HRESIMS analysis. Other than the signals from the *N*-carboxymethyl group (δ_C_ 168.5, δ_H_ 3.34 and 3.23), the ^1^H and ^13^C NMR spectra of this compound were very similar to those of **5**. The combined 2-D NMR analyses confirmed this similarity and **7**, designated pseudoaminol G, was determined to be a *N*-carboxymethyl-substituted amino alcohol.

The marine-derived amino alcohols and related metabolites exhibited cytotoxic [5,6,8,9,16] and antimicrobial activities [2,7,10,12,13,15,17]. In our cytotoxicity assays of the natural and synthetic derivatives, compounds **1**–**5**, **8**–**10**, and **12** showed cytotoxicity against the A549 and K562 cell lines that was substantially lower than that of doxorubicin, whereas the other compounds were inactive (Table 4). The significant cytotoxicity of the crude extract (LC_50_ 14.6 μg/mL against the A549 cell line) from this specimen suggests either the presence of minor but significantly cytotoxic constituents or the existence of synergic effects between these amino alcohols that may require further investigation.

In antibacterial assays against Gram-positive and Gram-negative strains, compounds **1**, **2**, **8**, and **9** exhibited moderate inhibition, whereas other compounds with more elaborate structures were inactive. These compounds were also tested against microbial enzymes, isocitrate lyase, sortase A, and Na^+^/K^+^-ATPase. Weak inhibition was observed for **1**, **2**, **8**, and **9** against enzyme Na^+^/K^+^-ATPase, whereas no other enzyme-inhibitory activities were observed for the amino alcohols. These results suggest that fewer double bonds and free amino and hydroxy groups are important for the antibacterial or related enzyme-inhibitory activity of the compounds. The absence of bioactivity for **6** and **7**, which possess the unprecedented *N*-carboxymethyl group, emphasizes the role the free amino group plays, via either an electronic or steric effect, in determining the bioactivity.

**Table 4:** The Result of Bioactivity test.

	LC_50_ (μM)		MIC (μg/mL)						IC_50_ (μM)
	K562	A549	Gram(+) Bacterium			Gram(−) Bacterium			Na^+^/K^+^-ATPase
Compound			A	B	C	D	E	F	
**1**	13.6	13.8	12.5	25	12.5	6.25	25	>100	62.0
**2**	12.6	13.1	12.5	25	12.5	12.5	25	>100	78.4
**3**	12.4	12.4	50	100	100	50	100	>100	>200
**4**	11.9	13.2	100	>100	100	50	>100	>100	190.3
**5**	>100	12.3	100	>100	100	50	>100	>100	101.2
**6**	>100	>100	>100	>100	>100	>100	>100	>100	>200
**7**	>100	>100	>100	>100	>100	>100	>100	>100	>200
**8**	12.7	10.5	50	50	25	12.5	50	>100	71.4
**9**	>100	14.0	12.5	25	12.5	6.25	25	>100	46.8
**10**	17.3	11.7	50	100	100	50	100	>100	118.1
**12**	5.9	8.0	>100	>100	>100	>100	>100	>100	108.2
**13**	>100	>100	100	>100	100	100	>100	>100	130.3
**14**	>100	>100	>100	>100	>100	>100	>100	>100	>200
Doxorubicin	1.1	0.9							
Ouabain									6.1
Ampicillin			0.4	0.4	0.4	0.4	1.6	6.3	

A: *Staphylococcus aureus* (ATCC 6538p); B: *Bacillus subtilis* (ATCC 6633), C: *Micrococcus luteus* (IFO 12708); D: *Salmonella typhimurium* (ATCC 14028); E: *Proteus vulgaris* (ATCC 3851); F: *Escherichia coli* (ATCC 35270).

## 3. Experimental Section

### 3.1. General Experimental Procedures

Optical rotations were measured on a JASCO P-1020 polarimeter (Jasco, Tokyo, Japan) using a 1 cm cell. UV spectra were acquired with a Hitachi U-3010 spectrophotometer (Hitachi High-Technologies, Tokyo, Japan). CD spectra were obtained on a JASCO J-715 (Jasco, Tokyo, Japan) using a 0.2 mm cell. IR spectra were recorded on a JASCO 4200 FT-IR spectrometer (Jasco, Tokyo, Japan) using a ZnSe cell. NMR spectra were recorded in DMSO-*d*_6_ and MeOH-*d*_4_ solutions containing Me_4_Si as an internal standard on Bruker Avance 600 and 500 spectrometers (Bruker, Massachusetts, MA, USA). Proton and carbon NMR spectra were measured at 600 and 150 MHz (**1**, **3**, **4**, **6**, and **7**) or 500 and 125 MHz (**2** and **5**), respectively (See Supplementary Information, Figures S3–S37). High-resolution FAB mass spectrometric data were obtained at the Korea Basic Science Institute (Daegu, Korea) and were acquired using a JEOL JMS 700 mass spectrometer (Jeol, Tokyo, Japan) with *meta*-nitrobenzyl alcohol (NBA) as a matrix for the FABMS. High-resolution ESIMS data were obtained at the National Instrumentation Center for Environmental Management (Seoul, Korea) using a Thermo-Finnigan LTQ-Orbitrap instrument (Thermo, Waltham, MA, USA) equipped with a Dionex U-3000 HPLC system (Thermo, Waltham, MA, USA). Low-resolution ESIMS data were recorded on an Agilent Technologies 6130 quadrupole mass spectrometer (Santa Clara, CA, USA) coupled to an Agilent Technologies 1200 series HPLC (Santa Clara, CA, USA). Semi-preparative HPLC was performed on a Spectrasystem p2000 (Thermo, Waltham, MA, USA) equipped with a refractive-index detector (Spectrasystem RI-150) and a YMC ODS-A column (10 × 250 mm). All solvents used were spectroscopic grade or were distilled prior to use.

### 3.2. Animal Materials

Specimens of *Pseudodistoma* sp. (sample number 12CH-24) were collected by hand using scuba equipment at a depth of 20 m off the coast of Chuja-do, Korea, on 10 October 2012. The colony has conical heads on short, thick, and wrinkled cylindrical stalks. Nine stalks (16–29 mm long) were on a basal test mass up to 62 mm wide. The colony was reddish-orange in live form and was yellowish-beige in ethanol. The zooids were reddish-orange in color during the tests. The zooids were 10.2–32.0 mm in length; the thorax was 3.5–4.2 mm, and the abdomen was 6.7–27.8 mm. These morphological features indicated that the specimen belongs to the genus *Pseudodistoma*, and in particular, was very similar to *P. antinboja* Tokioka; however, the lack of gonads and larvae prevented adequate species identification. The voucher specimens were deposited at the Natural History Museum, Ehwa Womans University, under the curatorship of B.J.R.

### 3.3. Extraction and Isolation

Freshly collected specimens were immediately frozen and stored at −25 °C until use. Lyophilized specimens were macerated and repeatedly extracted with MeOH (3 L × 3) and CH_2_Cl_2_ (3 L × 2). The combined extracts (25.71 g) were successively partitioned between H_2_O (18.67 g) and *n*-BuOH (7.05 g); the latter fraction was repartitioned between H_2_O–MeOH (15:85) (4.50 g) and *n*-hexane (2.55 g). The former layer was separated by C_18_ reversed-phase flash chromatography using sequential mixtures of MeOH and H_2_O as the eluents (six fractions in the gradient, H_2_O–MeOH, from 50:50 to 0:100), followed by acetone and finally EtOAc.

On the basis of the ^1^H NMR results and cytotoxicity analyses, the fractions eluted with 40:60 H_2_O–MeOH (0.88 g) and 20:80 H_2_O–MeOH (0.22 g) were chosen for separation. The 40:60 H_2_O–MeOH fraction (0.88 g) was separated by reversed-phase semi-preparative HPLC (H_2_O–MeOH, 50:50), yielding five peaks rich with secondary metabolites. The five peaks provided, in order of elution, compounds **5**, **3**, **4**, **1**, and **2**; the products were highly pure and required no further purification.

The 20:80 H_2_O–MeOH fraction (0.22 g) was separated by reversed-phase semi-preparative HPLC (H_2_O–MeOH, 30:70) to yield, in order of elution, compounds 6 and 7. The purified metabolites were isolated in the following amounts: 87.0, 75.3, 6.3, 3.3, 35.0, 3.9, and 4.1 mg of 1–7, respectively.

Pseudoaminol A (**1**): Yellow amorphous solid; 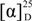 −4.4 (*c* 0.40, MeOH); IR (ZnSe) ν_max_ 3352, 2924, 1655 cm^−1^; ^1^H and ^13^C NMR data, see Table 1 and Table 2, respectively; HRFABMS *m*/*z* 228.2329 [M + H]^+^ (calcd for C_14_H_30_NO, 228.2327).

Pseudoaminol B (**2**): Yellow amorphous solid; 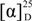 +5.1 (*c* 0.50, MeOH); IR (ZnSe) ν_max_ 3336, 2925, 1671 cm^−1^; ^1^H and ^13^C NMR data, see Table 1 and Table 2, respectively; HRFABMS *m*/*z* 228.2330 [M + H]^+^ (calcd for C_14_H_30_NO, 228.2327).

Pseudoaminol C (**3**): Yellow amorphous solid; 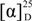 +2.7 (*c* 0.50, MeOH); UV (MeOH) λ_max_ (log ε) 204 (2.58), 228 (2.48) nm; IR (ZnSe) ν_max_ 3389, 2925, 1670 cm^−1^; ^1^H and ^13^C NMR data, see Table 1 and Table 2, respectively; HRFABMS *m*/*z* 226.2175 [M + H]^+^ (calcd for C_14_H_28_NO, 226.2171).

Pseudoaminol D (**4**): Yellow amorphous solid; 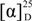 +15.9 (*c* 0.65, MeOH); IR (ZnSe) ν_max_ 3336, 2924, 1716, 1651 cm^−1^; ^1^H and ^13^C NMR data, see Table 1 and Table 3, respectively; HRFABMS *m*/*z* 214.2171 [M + H]^+^ (calcd for C_13_H_28_NO, 214.2171).

Pseudoaminol E (**5**): Yellow amorphous solid; 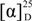 +2.4 (*c* 0.45, MeOH); UV (MeOH) λ_max_ (log ε) 205 (2.48), 230 (2.49), 278 (1.69) nm; IR (ZnSe) ν_max_ 3340, 2928, 1670 cm^−1^; ^1^H and ^13^C NMR data, see Table 1 and Table 3, respectively; HRFABMS *m*/*z* 250.2174 [M + H]^+^ (calcd for C_16_H_28_NO, 250.2171).

Pseudoaminol F (**6**): White amorphous solid; 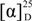 +3.4 (*c* 0.50, MeOH); IR (ZnSe) ν_max_ 3386, 2923, 1747, 1640 cm^−1^; ^1^H and ^13^C NMR data, see Table 1 and Table 3, respectively; HRFABMS *m*/*z* 286.2386 [M + H]^+^ (calcd for C_16_H_32_NO_3_, 286.2382).

Pseudoaminol G (**7**): White amorphous solid; 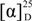 +1.4 (*c* 0.50, MeOH); UV (MeOH) λ_max_ (log ε) 204 (2.48), 232 (2.34), 275 (1.58) nm; IR (ZnSe) ν_max_ 3381, 2925, 1736, 1646 cm^−1^; ^1^H and ^13^C NMR data, see Table 1 and Table 3, respectively; HRESIMS *m*/*z* 308.2220 [M + H]^+^ (calcd for C_18_H_30_NO_3_, 308.2222).

### 3.4. Preparation of Oxazolidinone Derivative

To a stirred solution of 3.0 mg (0.013 mmol) of compound **1** in 2.0 mL of dry CH_2_Cl_2_ was added 15.0 mg of 1,1′-carbonyldiimidazole. The mixture was stirred at room temperature for 2 h, concentrated under reduced pressure, and purified by HPLC (YMC ODS-A column, 10 × 250 mm; H_2_O–MeOH, 20:80) to yield pure compound **8** (2.5 mg, 75%).

Compound 8 (**8**): ^1^H NMR (MeOH-*d*_4_) δ 5.86 (1H, dt, *J* = 15.0, 7.0 Hz, H-5), 5.53 (1H, dd, *J* = 15.0, 7.5 Hz, H-4), 4.45 (1H, dd, *J* = 7.5, 7.5 Hz, H-3), 3.59 (1H, dq, *J* = 7.5, 6.2 Hz, H-2), 2.08 (2H, dt, *J* = 7.0, 7.0 Hz, H-6), 1.24–1.39 (14H, m, H-7–H-13), 0.88 (3H, t, *J* = 7.0 Hz, H-14); LRESIMS *m*/*z* 254.2 [M + H]^+^ (calcd for C_15_H_28_NO_2_, 254.2).

### 3.5. Hydrogenation of Amino Alcohols

To a stirred mixture of 8.7 mg (0.038 mmol) of **1** in 1.0 mL of dry MeOH was added 10.0 mg of Pd/C. The mixture was stirred and hydrogenated using H_2_ at room temperature for 2 h. The mixture was filtered through Celite under vacuum. The residue was concentrated under reduced pressure and purified by HPLC (YMC ODS-A column, 10 × 250 mm; H_2_O–MeOH, 50:50) to yield pure compound **9** (7.1 mg, 81%). Compound **4** (2.8 mg, 0.013 mmol) was hydrogenated to afford pure compound **13** (2.3 mg, 81%) via the same procedure as that for **1**.

Compound 9 (**9**): ^1^H NMR (MeOH-*d*_4_) δ 3.20 (1H, m, H-3), 2.60 (1H, dq, *J* = 7.0, 7.0 Hz, H-2), 1.49 (2H, m, H-4), 1.23–1.38 (18H, m, H-5–H-13), 1.04 (3H, d, *J* = 7.0 Hz, H-1), 0.89 (3H, t, *J* = 7.0 Hz, H-14); LRESIMS *m*/*z* 230.3 [M + H]^+^ (calcd for C_14_H_32_NO, 230.3).

Compound 13 (**13**): ^1^H NMR (MeOH-*d*_4_) δ 3.62 (1H, m, H-2), 2.85 (1H, m, H-1a), 2.63 (1H, m, H-1b), 1.44 (2H, m, H-3), 1.22–1.36 (12H, m, H-4–H-12), 0.88 (3H, t, *J* = 7, H-13); LRESIMS *m*/*z* 215.2 [M + H]^+^ (calcd for C_13_H_30_NO, 215.2).

### 3.6. Preparation of N-Acetyl Derivatives

To a stirred mixture of 2.9 mg (0.013 mmol) of **9** in 1.0 mL of dry pyridine was added 5.0 mg of acetic anhydride. The mixture was stirred at room temperature for 1 h, concentrated under reduced pressure, and purified by HPLC (YMC ODS-A column, 10 × 250 mm; H_2_O–MeOH, 20:80) to yield pure compound **10** (2.3 mg, 71%). Compound **13** (2.3 mg, 0.011 mmol) was acetylated to afford pure compound **14** (1.9 mg, 77%) via the same procedure as that for **9**.

Compound 10 (**10**): ^1^H NMR (CDCl_3_) δ 5.86 (1H, s, NH), 3.70 (1H, m, H-2), 3.51 (1H, m, H-1), 3.10 (1H, m, H-1), 2.04 (3H, s, H-NHCOMe-Me), 1.44 (2H, m, H-3), 1.24–1.33 (12H, m, H-7–H-12), 0.88 (3H, t, *J* = 7, H-13); LRESI-MS *m*/*z* 258.2 [M + H]^+^ (calcd for C_15_H_32_NO_2_, 258.2).

Compound 14 (**14**): ^1^H NMR (CDCl_3_) δ 5.83 (1H, s, NH), 3.69 (1H, m, H-2), 3.52 (1H, m, H-1), 3.13 (1H, m, H-1), 2.01 (3H, s, H-NHCOMe-Me), 1.43 (1H, m, H-3), 1.24–1.30 (18H, m, H-4–H-12), 0.88 (3H, t, *J* = 7.5, H-13); LRESI-MS *m*/*z* 256.3 [M + H]^+^ (calcd for C_15_H_30_NO_2_, 256.2).

### 3.7. Esterification with of (–)-(R)-α-Methoxy-α-(trifluoromethyl)phenylacetic (MTPA) Chloride

To a stirred mixture of 1.0 mg (0.004 mmol) of **9** and 0.1 mg of DMAP in 1.0 mL of dry pyridine was added 20 μL of (–)-(*R*)-MTPA-chloride. The mixture was stirred at room temperature for 1 h. The mixture was concentrated under reduced pressure and purified by HPLC (YMC ODS-A column, 10 × 250 mm, H_2_O–MeOH, 10:90) to give 0.7 mg (32%) of (*S*)-MTPA ester **11S**. The corresponding (*R*)-MTPA ester **11R** (0.8 mg, 36%) was also obtained from a similar esterification reaction of **9** (1.0 mg, 0.004 mmol) with (+)-(*S*)-MTPA-chloride. Compound **14** (1.0 mg, 0.004 mmol) was esterified to afford pure compounds **15S** (0.6 mg, 32%) and **15R** (0.6 mg, 32%) via the same procedure as that for **9**.

Compound 11S (**11S**): ^1^H NMR (CDCl_3_) δ 7.508 (2H, dd, *J* = 7.6, 1.8 Hz, MTPA-Ar), 7.396–7.421 (3H, m, MTPA-Ar), 6.916 (1H, d, *J* = 9.0 Hz, NH), 4.920 (1H, dt, *J* = 5.0, 7.0 Hz, H-3), 4.254 (1H, ddq, *J* = 9.0, 5.0, 7.0 Hz, H-2), 3.408 (3H, s, MTPA-OMe), 2.092 (3H, s, COMe), 1.561 (2H, dt, *J* = 7.0, 7.0 Hz, H-4), 1.205–1.314 (18H, m, H-5–H-13), 1.119 (3H, d, *J* = 7.0 Hz, H-1), 0.880 (3H, t, *J* = 7.0 Hz, H-14); LRESIMS *m*/*z* 510.3 [M + Na]^+^ (calcd for C_26_H_40_NO_4_F_3_Na, 510.3).

Compound 11R (**11R**): ^1^H NMR (CDCl_3_) δ 7.545 (2H, dd, *J* = 7.5, 2.0 Hz, MTPA-Ar), 7.348–7.442 (3H, m, MTPA-Ar), 6.830 (1H, d, *J* = 9.0 Hz, NH), 4.880 (1H, dt, *J* = 5.0, 7.0 Hz, H-3), 4.239 (1H, ddq, *J* = 9.0, 5.0, 7.0 Hz, H-2), 3.437 (3H, s, MTPA-OMe), 1.969 (3H, s, COMe), 1.435 (2H, dt, *J* = 7.0, 7.0 Hz, H-4), 1.202–1.316 (18H, m, H-5–H-13), 1.171 (3H, d, *J* = 7.0 Hz, H-1), 0.880 (3H, t, *J* = 7.0 Hz, H-14); LRESIMS *m*/*z* 510.3 [M + Na]^+^ (calcd for C_26_H_40_NO_4_F_3_Na, 510.3).

Compound 15S (**15S**): ^1^H NMR (CDCl_3_) δ 7.497–7.540 (2H, m, MTPA-Ar), 7.377–7.418 (3H, m, MTPA-Ar), 7.035 (1H, dd, *J* = 5.5, 7.0 Hz, NH), 4.961 (1H, ddt, *J* = 3.5, 7.0, 7.0 Hz, H-2), 3.542 (1H, ddd, *J* = 3.5, 5.5, 13.5 Hz, H-1), 3.462 (1H, ddd, *J* = 7.0, 7.0, 13.5 Hz, H-1), 3.395 (3H, s, MTPA-OMe), 2.023 (3H, s, COMe), 1.299 (2H, m, H-3), 1.215–1.304 (18H, m, H-4–H-12), 0.880 (3H, t, *J* = 7.0 Hz, H-13); LRESIMS *m*/*z* 474.2 [M + H]^+^ (calcd for C_25_H_39_NO_4_F_3_, 474.3).

Compound 15R (**15R**): ^1^H NMR (CDCl_3_) δ 7.510–7.564 (2H, m, MTPA-Ar), 7.385–7.451 (3H, m, MTPA-Ar), 5.212 (1H, dd, *J* = 5.5, 7.0 Hz, NH), 5.121 (1H, ddt, *J* = 3.5, 7.0, 7.0 Hz, H-2), 3.602 (1H, ddd, *J* = 3.5, 5.5, 13.5 Hz, H-1), 3.566 (3H, s, MTPA-OMe), 3.246 (1H, ddd, *J* = 7.0, 7.0, 13.5 Hz, H-1), 1.839 (3H, s, COMe), 1.669 (2H, m, H-3), 1.214–1.315 (18H, m, H-4–H-12), 0.880 (3H, t, *J* = 7.0 Hz, H-13); LRESIMS *m*/*z* 474.2 [M + H]^+^ (calcd for C_25_H_39_NO_4_F_3_, 474.3).

### 3.8. Preparation of Dibenzoyl Derivatives

To a stirred mixture of 2.7 mg (0.012 mmol) of **9** and 0.1 mg of DMAP in 1.0 mL of dry pyridine was added 20.0 mg of benzoyl chloride. The mixture was stirred at room temperature for 3 h, concentrated under reduced pressure, and separated by HPLC (YMC ODS-A column, 10 × 250 mm; H_2_O–MeOH, 10:90) to yield compound **12** (2.1 mg, 41%). Compound **4** (2.5 mg, 0.012 mmol) was reacted to afford pure compound **16** (2.3 mg, 46%) via the same procedure as that for **9**.

Compound 12 (**12**): ^1^H NMR (CDCl_3_) δ 8.03 (2H, d, *J* = 7.8 Hz, Ar), 7.72 (2H, d, *J* = 7.8 Hz, Ar), 7.54 (1H, t, *J* = 7.8 Hz, Ar), 7.45 (5H, m, Ar), 6.38 (1H, d, *J* = 9.2 Hz, NH), 5.22 (1H, dt, *J* = 7.5, 5.5 Hz, H-3), 4.54 (1H, ddq, *J* = 7.5, 9.2, 7.0 Hz, H-2), 1.78 (1H, dddd, *J* = 7.5, 7.5, 7.5, 15.0 Hz, H-4), 1.78 (1H, dddd, *J* = 5.5, 7.5, 7.5, 15.0 Hz, H-4), 1.41 (2H, tt, *J* = 7.5, 7.5 Hz, H-5), 1.29 (3H, d, *J* = 7.0 Hz, H-1), 1.19–1.25 (16 H, m, H-6–H-13), 0.87 (3H, t, *J* = 7.0 Hz, H-14); LRESIMS *m*/*z* 438.3 [M + H]^+^ (calcd for C_28_H_40_NO_3_, 438.3).

Compound 16 (**16**): ^1^H NMR (CDCl_3_) δ 8.05 (2H, d, *J* = 7.9 Hz, Ar), 7.74 (2H, d, *J* = 7.9 Hz, Ar), 7.58 (1H, t, *J* = 7.9 Hz, Ar), 7.45 (5H, m, Ar), 6.69 (1H, dd, *J* = 5.5, 5.5 Hz, NH), 5.60 (1H, ddd, *J* = 14.5, 7.5, 7.5 Hz, H-5), 5.45 (1H, ddd, *J* = 14.5, 7.5, 7.5 Hz, H-4), 5.29 (1H, dddd, *J* = 7.5, 7.5, 7.5, 3.0 Hz, H-2), 3.83 (1H, ddd, *J* = 13.5, 5.5, 3.0 Hz, H-1), 3.72 (1H, ddd, *J* = 13.5, 7.5, 5.5 Hz, H-1), 2.50 (2H, dd, *J* = 7.5, 7.5 Hz, H-3), 1.98 (2H, dt, *J* = 7.5, 7.5 Hz, H-6), 1.20–1.31 (12H, m, H-7–H-12), 0.87 (3H, t, *J* = 7.0 Hz, H-13); LRESIMS *m*/*z* 422.2 [M + H]^+^ (calcd for C_27_H_36_NO_3_, 422.3).

### 3.9. Biological Assays

Antimicrobial assays were performed according to the method described previously [22]. Cytotoxicity assays were performed in accord with literature protocols [23]. Isocitrate lyase, sortase A, and Na^+^/K^+^-ATPase inhibition assays were performed according to previously described methods [24,25,26].

## 4. Conclusions

Seven new amino alcohol compounds, pseudoaminols A–G (**1**–**7**), were isolated from the Korean ascidian *Pseudodistoma* sp. Structures of these new compounds were determined by analysis of the spectroscopic data and from diverse chemical conversion.

## Supplementary Files

Supplementary File 1 Supplementary Information (PDF, 2622 KB)

## References

[1] Blunt J.W., Copp B.R., Keyzers R.A., Munro M.H.G., Prinsep M.R. (2014). Marine natural products. Nat. Prod. Rep..

[2] Gulavita N.K., Scheuer P.J. (1989). Two epimeric aliphatic amino alcohols from a sponge, *Xestospongia* sp. J. Org. Chem..

[3] Jares-Erijman E.A., Bapat C.P., Lithgow-Bertelloni A., Rinehart K.L., Sakai R. (1993). Crucigasterins, new polyunsaturated amino alcohols from the Mediterranean tunicate *Pseudodistoma crucigaster*. J. Org. Chem..

[4] Hooper G.J., Davies-coleman M.T., Coetzee P.S. (1995). New antimicrobial C_14_ and C_13_ amines from a South African marine ascidian. Nat. Prod. Lett..

[5] Sata N.U., Fusetani N. (2000). Amaminols A and B, new bicyclic amino alcohols from an unidentified tunicate of the family Polyclinidae. Tetrahedron Lett..

[6] Garrido L., Zubía E., Ortega M.J., Naranjo S., Salvá J. (2001). Obscuraminols, new unsaturated amino alcohols from the tunicate *Pseudodistoma obscurum*: Structure and absolute configuration. Tetrahedron.

[7] Kossuga M.H., MacMillan J.B., Rogers E.W., Molinski T.F., Nascimento G.G.F., Rocha R.M., Berlinck R.G.S. (2004). (2*S*,3*R*)-2-Aminododecan-3-ol, a new antifungal agent from the ascidian *Clavelina oblonga*. J. Nat. Prod..

[8] Aiello A., Fattorusso E., Giordano A., Menna M., Navarrete C., Muñoz E. (2007). Clavaminols A–F, novel cytotixic 2-amino-3-alkanols from the ascidian *Clavelina phlegraea*. Bioorg. Med. Chem..

[9] Aiello A., Fattorusso E., Giordano A., Menna M., Navarrete C., Muñoz E. (2009). Clavaminols G–N, six new marine sphingoids from the Mediterranean ascidian *Clavelina phlegraea*. Tetrahedron.

[10] Ciavatta M.L., Manzo E., Nuzzo G., Villani G., Varcamonti M., Gavagnin M. (2010). Crucigasterins A–E, antimicrobial amino alcohols from the Mediterranean colonial ascidian *Pseudodistoma crucigaster*. Tetrahedron.

[11] Makarieva T.N., Denisenko V.A., Stonik V.A., Milgrom Y.M., Rashkes Y.V. (1989). Rhizochalin, a novel secondary metabolite of mixed biosynthesis from the sponge *Rhizochalina incrustata*. Tetrahedron Lett..

[12] Jimenez C., Crews P. (1990). Novel marine sponge amino acids, 10. Xestoaminols from *Xestospongia* sp. J. Nat. Prod..

[13] Kong F., Faulkner D.J. (1993). Leucettaminols A and B, two antimicrobial lipids from the calcareous sponge *Leucetta microraphis*. J. Org. Chem..

[14] Devijver C., Salmoun M., Daloze D., Breakman J.C., de Weerdt W.H., de Kluijver M.J., Gomez R. (2000). (2*R*,3*R*,7*Z*)-2-Aminotetradec-7-ene-1,3-diol, a new amino alcohol from the Caribbean sponge *Haliclona vansoeti*. J. Nat. Prod..

[15] Clark R.J., Garson M.J., Hooper J.N.A. (2001). Antifungal alkyl amino alcohols from the tropical sponge *Haliclona* n. sp. J. Nat. Prod..

[16] Cuadros R., de Garcini E. M., Wandosell F., Faircloth G., Fernández-Sousa J.M., Avila J. (2000). The marine compound spisulosine, an inhibitor of cell proliferation, promotes the disassembly of actin stress fibers. Cancer Lett..

[17] Ui H., Shiomi K., Suzuki H., Hatano H., Morimoto H., Yamaguchi Y., Masuma R., Sakamoto K., Kita K., Miyoshi H. (2006). Paecilaminol, a new NADH-fumarate reductase inhibitor, produced by *Paecilomyces* sp. FKI-0550. J. Antibiot..

[18] Hannun Y.A. (1994). The sphingomyelin cycle and the second messenger function of ceramide. J. Biol. Chem..

[19] Polt R., Sames D., Chruma J. (1999). Glycosidase inhibitors: Synthesis of enantiomerically pure aza-sugars from Schiff base amino esters via tandem reduction-alkenylation and osmylation. J. Org. Chem..

[20] Searle P.A., Molinski T.F. (1993). Structure and absolute configuration of (*R*)-(*E*)-1-aminotridec-5-en-2-ol, an antifungal amino alcohol from the ascidian *Didemnum* sp. J. Org. Chem..

[21] Wenkert E., Buckwalter B.L., Burfitt I.R., Gasic M.J., Gottlieb H.E., Hagaman E.W., Schell F.M., Wovkulich P.M., Levy G.C. (1976). Carbon-13 nuclear magnetic resonance spectroscopy of naturally occurring substances. Topics in Carbon-13 NMR Spectroscopy.

[22] Oh K.-B., Lee J.H., Chung S.-C., Shin J., Shin H.J., Kim H.-K., Lee H.-S. (2008). Antimicrobial activities of the bromophenols from the red alga *Odonthalia corymbifera* and some synthetic derivatives. Bioorg. Med. Chem. Lett..

[23] Van le T.K., Hung T.M., Thuong P.T., Ngoc T.M., Kim J.C., Jang H.-S., Cai X.F., Oh S.R., Min B.-S., Woo M.H. (2009). Oleanane-type triterpenoids from *Aceriphyllum rossii* and their cytotoxic activity. J. Nat. Prod..

[24] Johansson M., Karlsson L., Wennergren M., Jansson T., Powell T.L. (2003). Activity and protein expression of Na^+^/K^+^ ATPase are reduced in microvillous syncytiotrophoblast plasma membranes isolated from pregnancies complicated by intrauterine growth restriction. J. Clin. Endocrinol. Metab..

[25] Oh K.-B., Kim S.-H., Lee J., Cho W.-J., Lee T., Kim S. (2004). Discovery of diarylacrylonitriles as a novel series of small molecule sortase A inhibitors. J. Med. Chem..

[26] Chung S.C., Jang K.H., Park J., Ahn C.-H., Shin J., Oh K.-B. (2011). Actin depolymerizing effect of trisoxazole-containing macrolides. Bioorg. Med. Chem. Lett..

