# Supplementary material for: Amino Alcohols from the Ascidian Pseudodistoma sp
Source: Mar Drugs. 2014 Jun 24;12(6):3754–69. doi: 10.3390/md12063754 (PMC4071600; doi:10.3390/md12063754)
Supplement: Supplementary File 1 — Supplementary Information (PDF, 2622 KB) [file marinedrugs-12-03754-s001.pdf]

Supplementary Information

Figure S1. The CD spectra of the dibenzoyl derivatives (**12**) of **1** and **2**.
Figure S2. The CD spectra of the dibenzoyl derivative (**16**) of **4**.
Figure S3. The ^{1}H NMR (600 MHz, MeOH-d_4) spectrum of pseudoaminol A (**1**).
Figure S4. The ^{13}C NMR (150 MHz, MeOH-d_4) spectrum of pseudoaminol A (**1**).
Figure S5. The COSY (600 MHz, MeOH-d_4) spectrum of pseudoaminol A (**1**).
Figure S6. The *g*HSQC (600 MHz, MeOH-d_4) spectrum of pseudoaminol A (**1**).
Figure S7. The *g*HMBC (600 MHz, MeOH-d_4) spectrum of pseudoaminol A (**1**).
Figure S8. The ^{1}H NMR (500 MHz, MeOH-d_4) spectrum of pseudoaminol B (**2**).
Figure S9. The ^{13}C NMR (125 MHz, MeOH-d_4) spectrum of pseudoaminol B (**2**).
Figure S10. The COSY (500 MHz, MeOH-d_4) spectrum of pseudoaminol B (**2**).
Figure S11. The *g*HSQC (500 MHz, MeOH-d_4) spectrum of pseudoaminol B (**2**).
Figure S12. The *g*HMBC (500 MHz, MeOH-d_4) spectrum of pseudoaminol B (**2**).
Figure S13. The ^{1}H NMR (600 MHz, MeOH-d_4) spectrum of pseudoaminol C (**3**).
Figure S14. The ^{13}C NMR (150 MHz, MeOH-d_4) spectrum of pseudoaminol C (**3**).
Figure S15. The COSY (600 MHz, MeOH-d_4) spectrum of pseudoaminol C (**3**).
Figure S16. The *g*HSQC (600 MHz, MeOH-d_4) spectrum of pseudoaminol C (**3**).
Figure S17. The *g*HMBC (600 MHz, MeOH-d_4) spectrum of pseudoaminol C (**3**).
Figure S18. The ^{1}H NMR (600 MHz, MeOH-d_4) spectrum of pseudoaminol D (**4**).
Figure S19. The ^{13}C NMR (150 MHz, MeOH-d_4) spectrum of pseudoaminol D (**4**).
Figure S20. The COSY (600 MHz, MeOH-d_4) spectrum of pseudoaminol D (**4**).
Figure S21. The *g*HSQC (600 MHz, MeOH-d_4) spectrum of pseudoaminol D (**4**).
Figure S22. The *g*HMBC (600 MHz, MeOH-d_4) spectrum of pseudoaminol D (**4**).
Figure S23. The ^{1}H NMR (500 MHz, MeOH-d_4) spectrum of pseudoaminol E (**5**).
Figure S24. The ^{13}C NMR (125 MHz, MeOH-d_4) spectrum of pseudoaminol E (**5**).
Figure S25. The COSY (500 MHz, MeOH-d_4) spectrum of pseudoaminol E (**5**).
Figure S26. The *g*HSQC (500 MHz, MeOH-d_4) spectrum of pseudoaminol E (**5**).
Figure S27. The *g*HMBC (500 MHz, MeOH-d_4) spectrum of pseudoaminol E (**5**).
Figure S28. The ^{1}H NMR (600 MHz, DMSO-d_6) spectrum of pseudoaminol F (**6**).
Figure S29. The ^{13}C NMR (150 MHz, DMSO-d_6) spectrum of pseudoaminol F (**6**).
Figure S30. The COSY (600 MHz, DMSO-d_6) spectrum of pseudoaminol F (**6**).
Figure S31. The *g*HSQC (600 MHz, DMSO-d_6) spectrum of pseudoaminol F (**6**).
Figure S32. The *g*HMBC (600 MHz, DMSO-d_6) spectrum of pseudoaminol F (**6**).
Figure S33. The ^{1}H NMR (600 MHz, DMSO-d_6) spectrum of pseudoaminol G (**7**).
Figure S34. The ^{13}C NMR (150 MHz, DMSO-d_6) spectrum of pseudoaminol G (**7**).
Figure S35. The COSY (600 MHz, DMSO-d_6) spectrum of pseudoaminol G (**7**).
Figure S36. The *g*HSQC (600 MHz, DMSO-d_6) spectrum of pseudoaminol G (**7**).
Figure S37. The *g*HMBC (600 MHz, DMSO-d_6) spectrum of pseudoaminol G (**7**).

Figure S1. CD spectra of the dibenzoyl derivatives (**12**) of **1** and **2**.

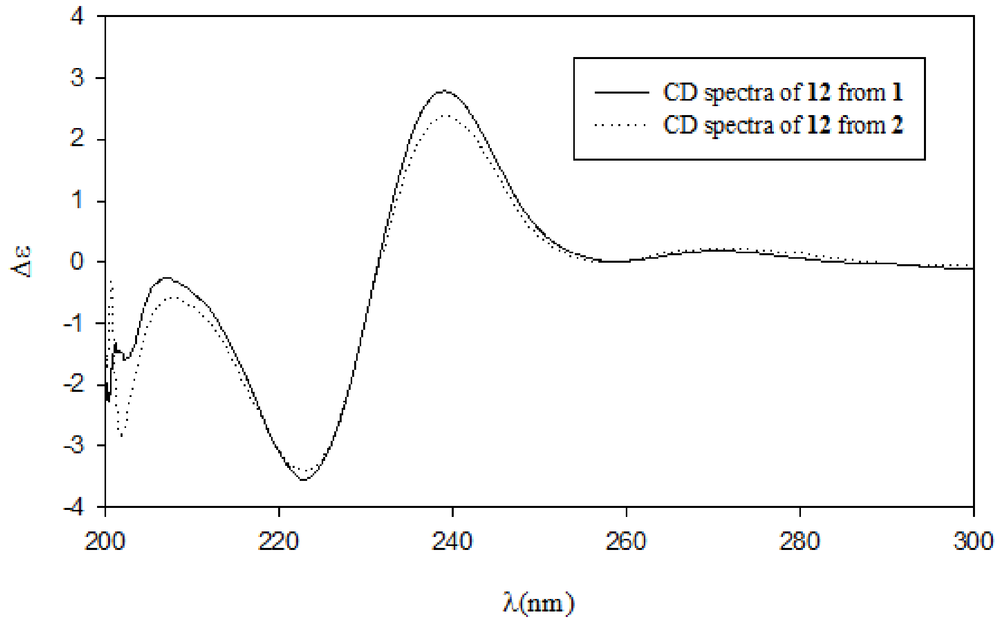

Figure S2. The CD spectra of the dibenzoyl derivative (**16**) of **4**.

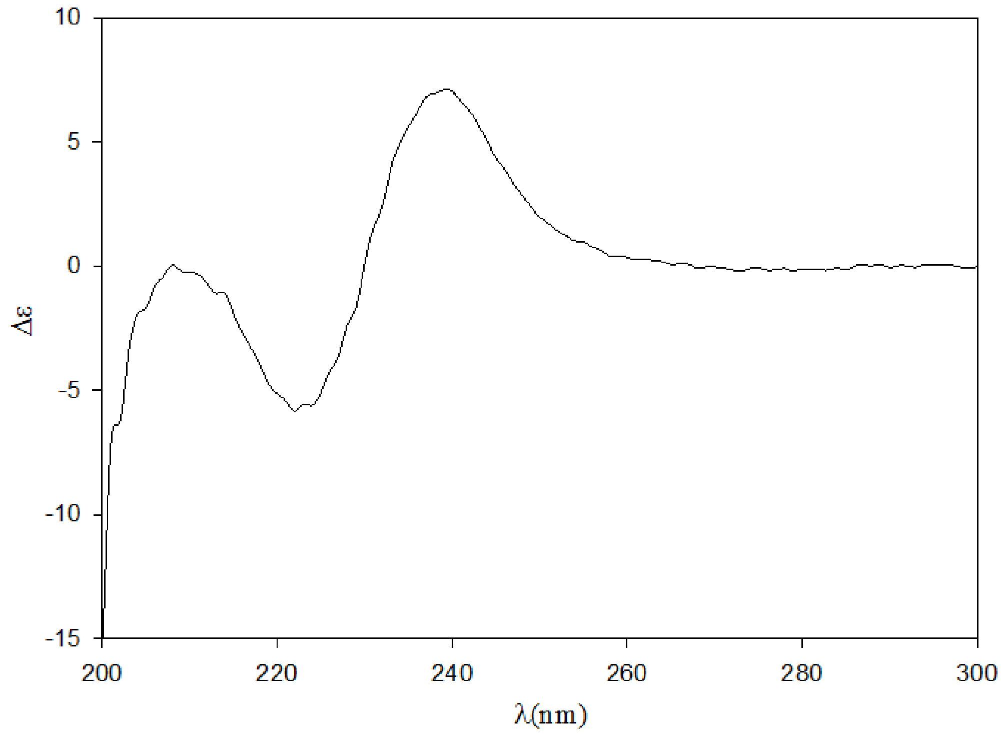

Figure S3. The ^{1}H NMR (600 MHz, MeOH-d_4) spectrum of pseudoaminol A (**1**).

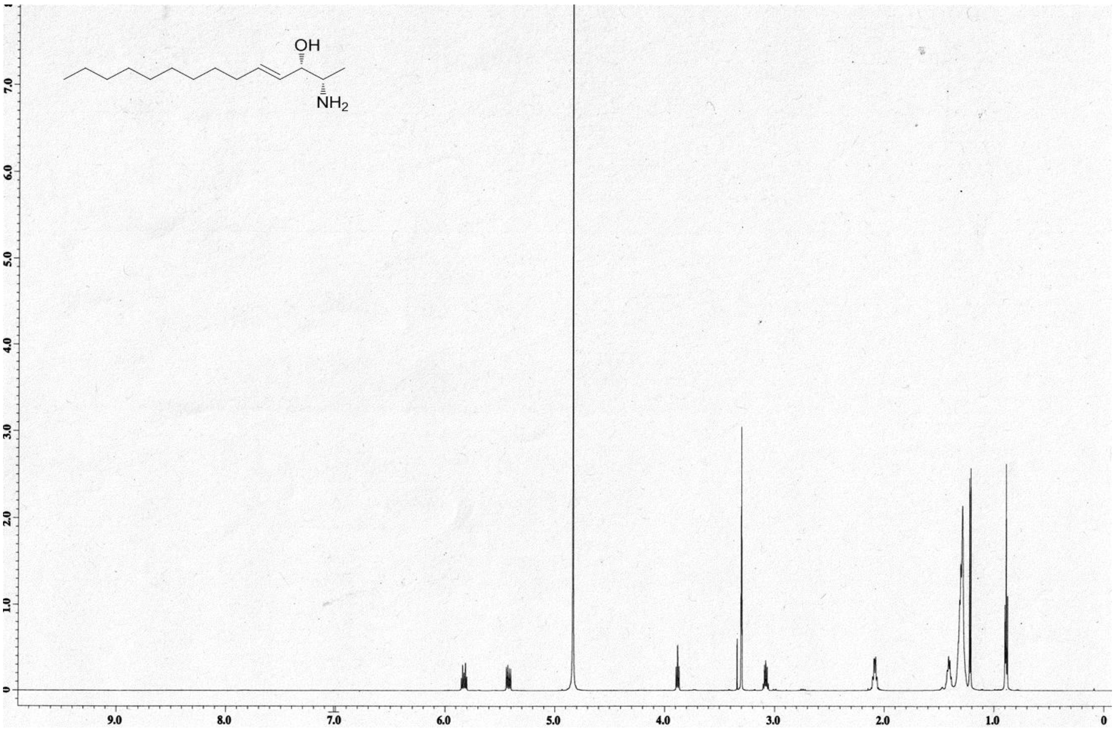

Figure S4. The ^{13}C NMR (150 MHz, MeOH-d_4) spectrum of pseudoaminol A (**1**).

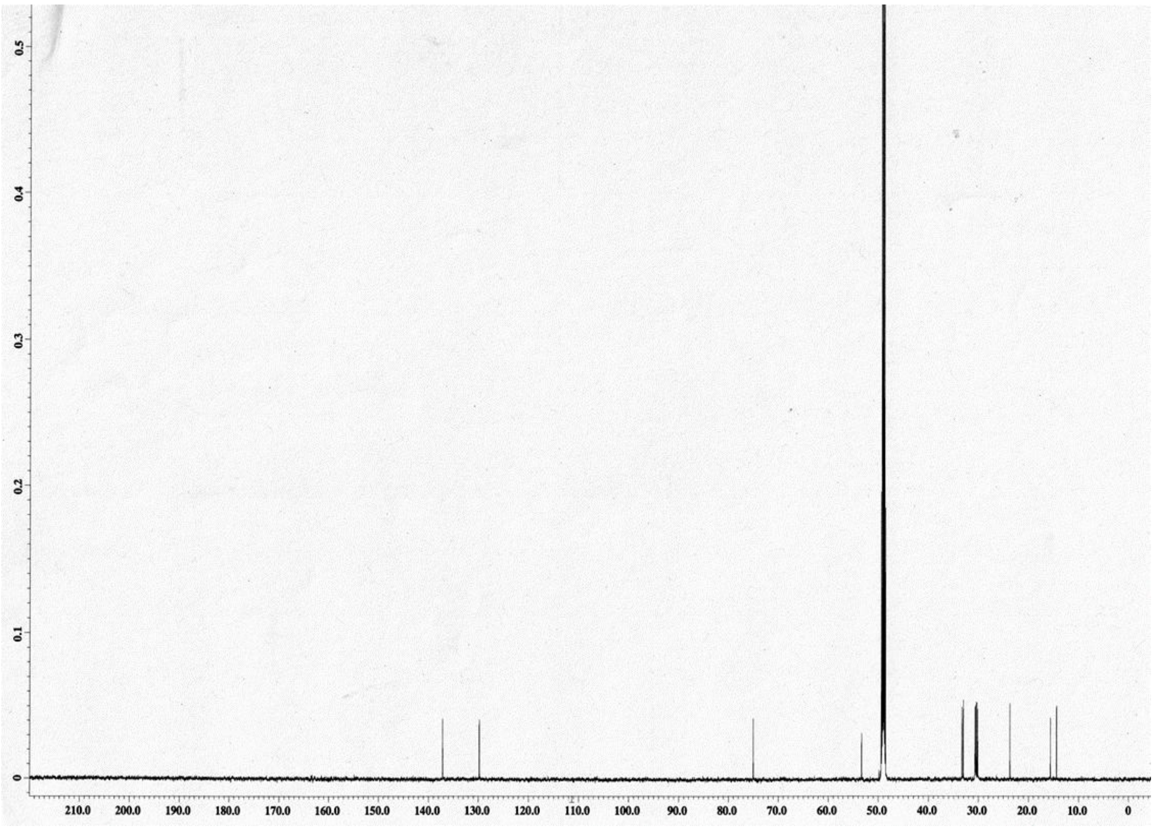

Figure S5. The COSY (600 MHz, MeOH-d_4) spectrum of pseudoaminol A (**1**).

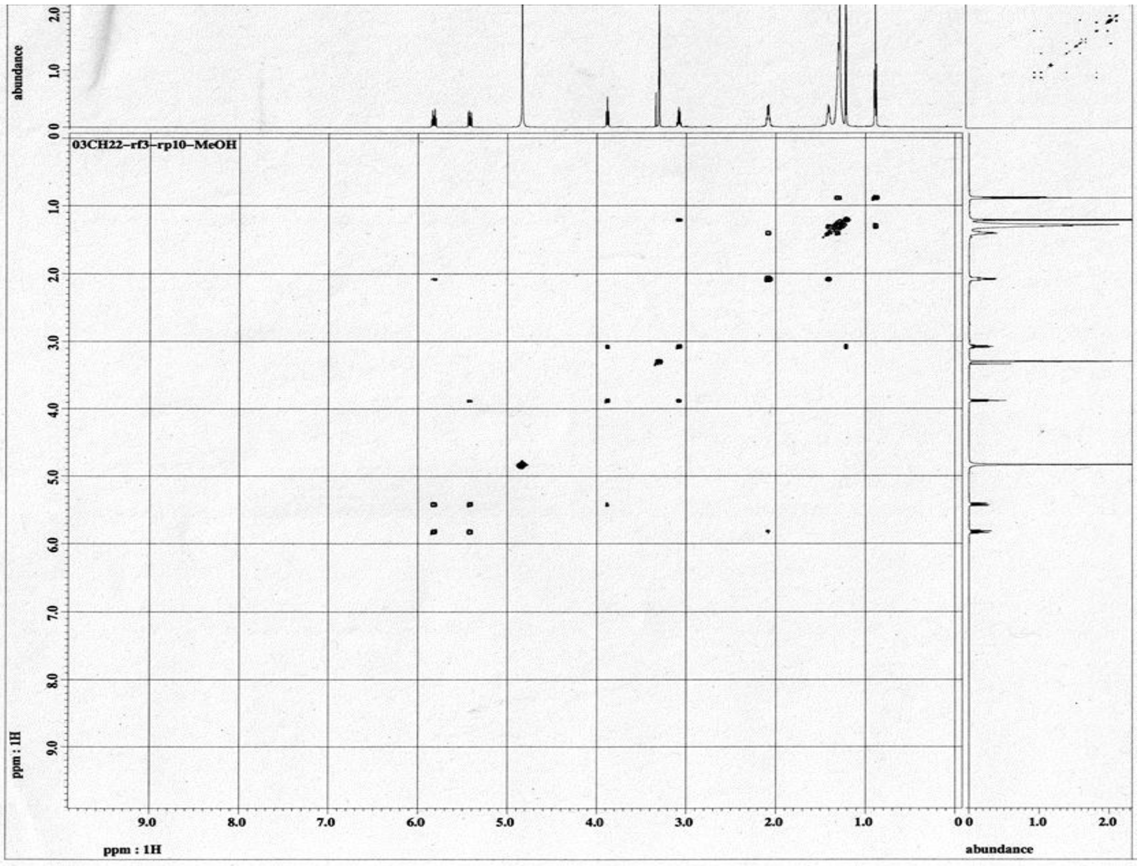

Figure S6. The *g*HSQC (600 MHz, MeOH-d_4) spectrum of pseudoaminol A (**1**).

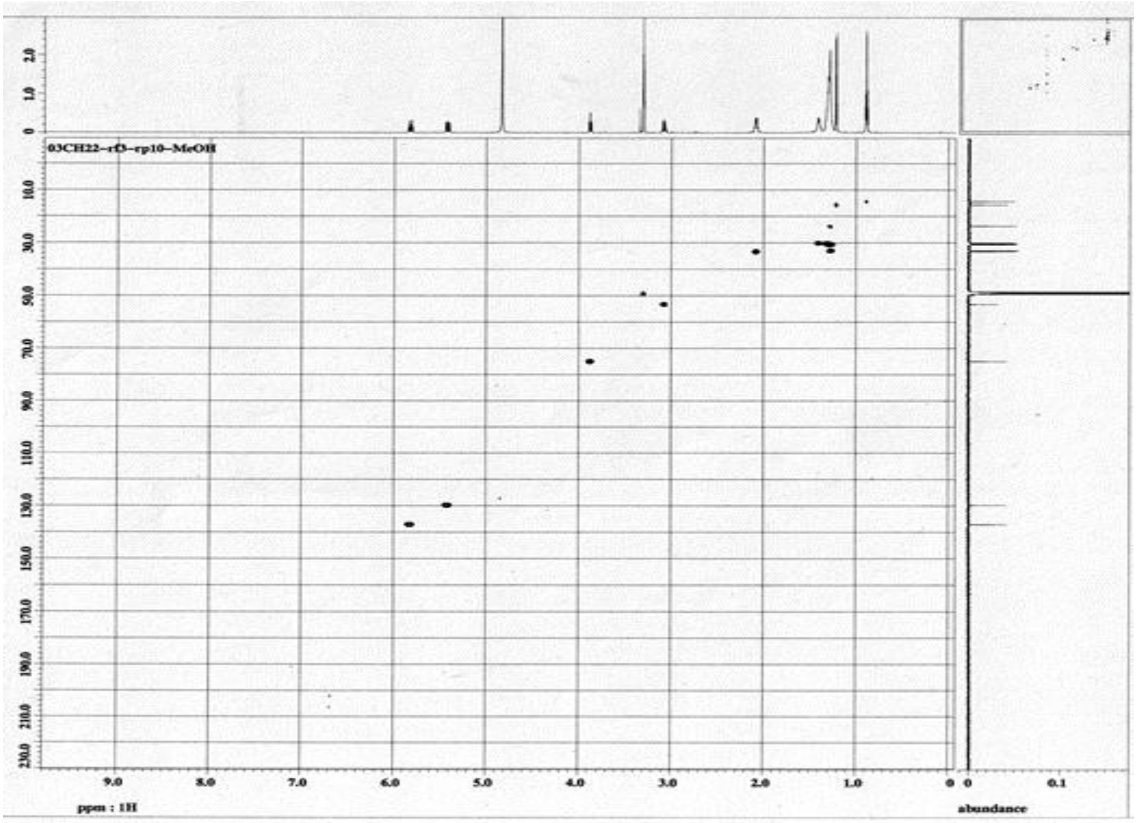

Figure S7. The *g*HMBC (600 MHz, MeOH-d_4) spectrum of pseudoaminol A (**1**).

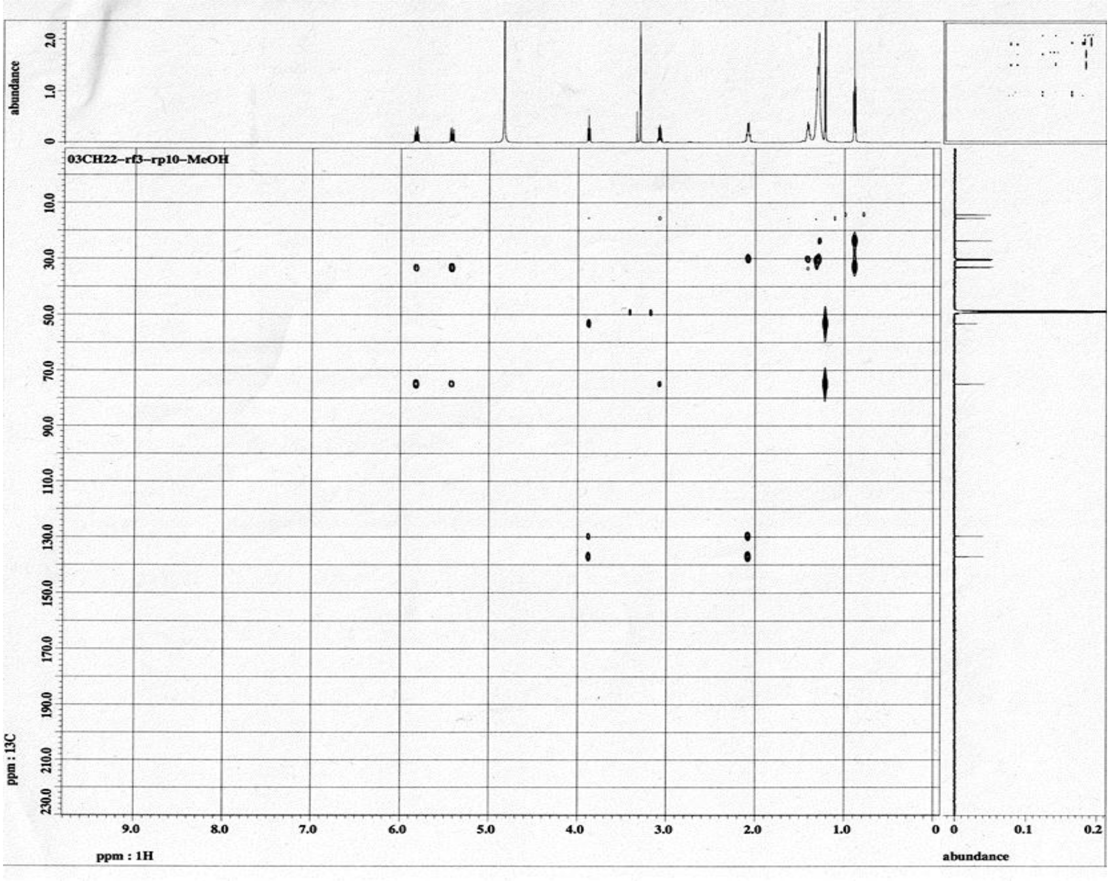

Figure S8. The ^{1}H NMR (500 MHz, MeOH-d_4) spectrum of pseudoaminol B (**2**).

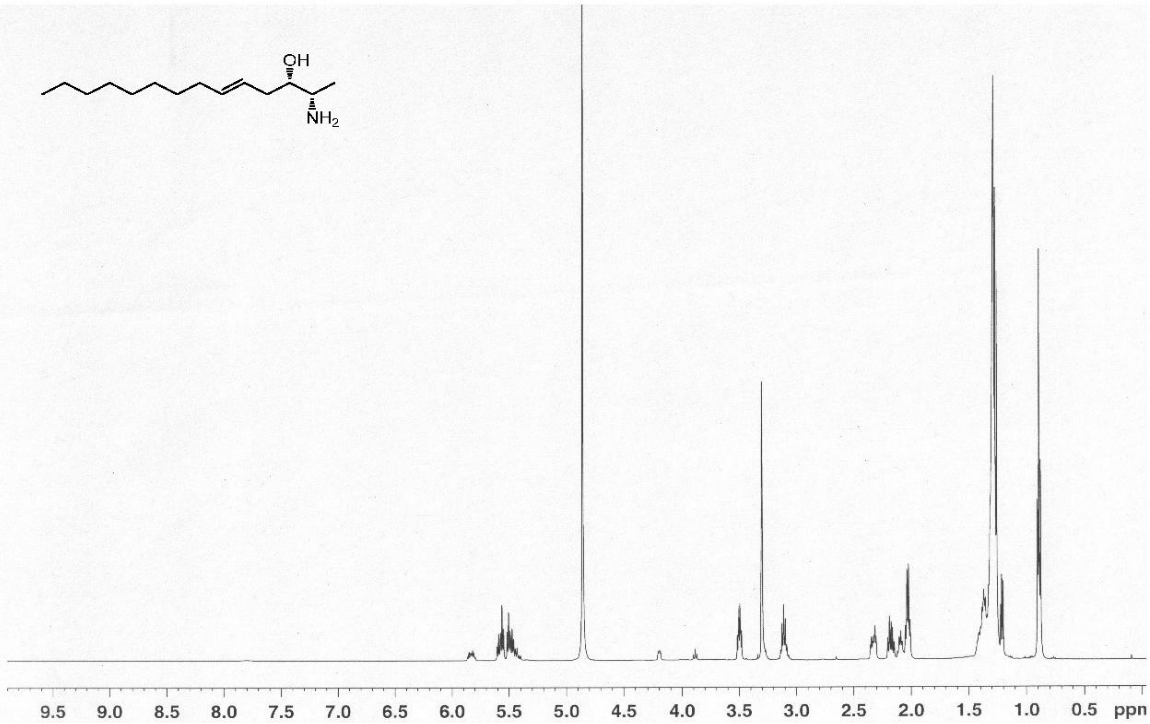

Figure S9. The ^{13}C NMR (125 MHz, MeOH-d_4) spectrum of pseudoaminol B (**2**).

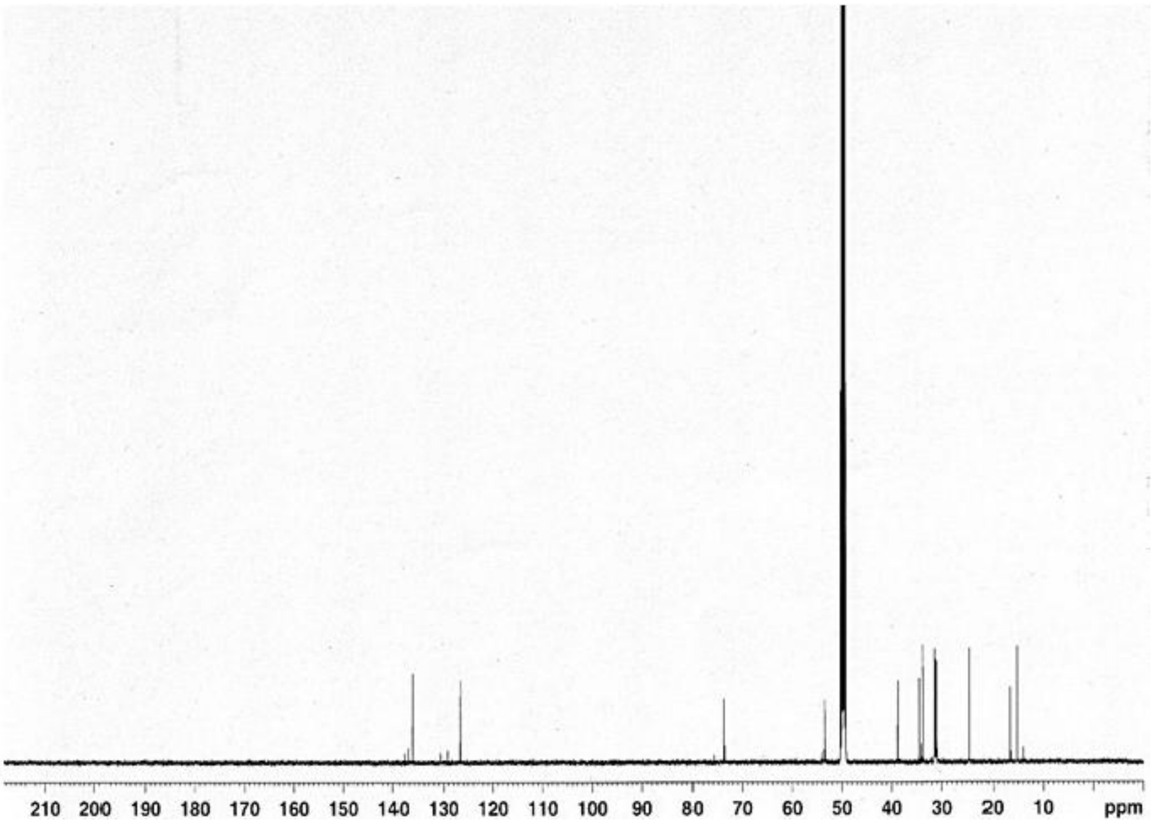

Figure S10. The COSY (500 MHz, MeOH-d_4) spectrum of pseudoaminol B (**2**).

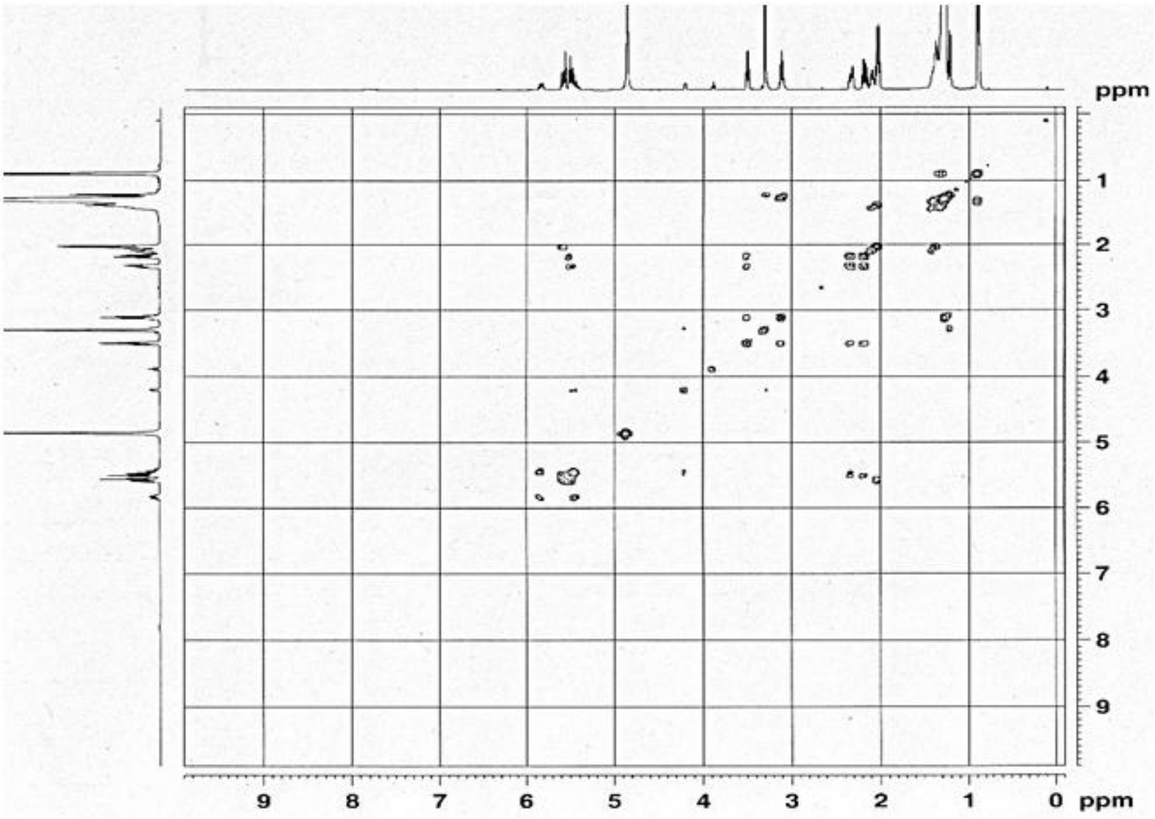

Figure S11. The *g*HSQC (500 MHz, MeOH-d_4) spectrum of pseudoaminol B (**2**).

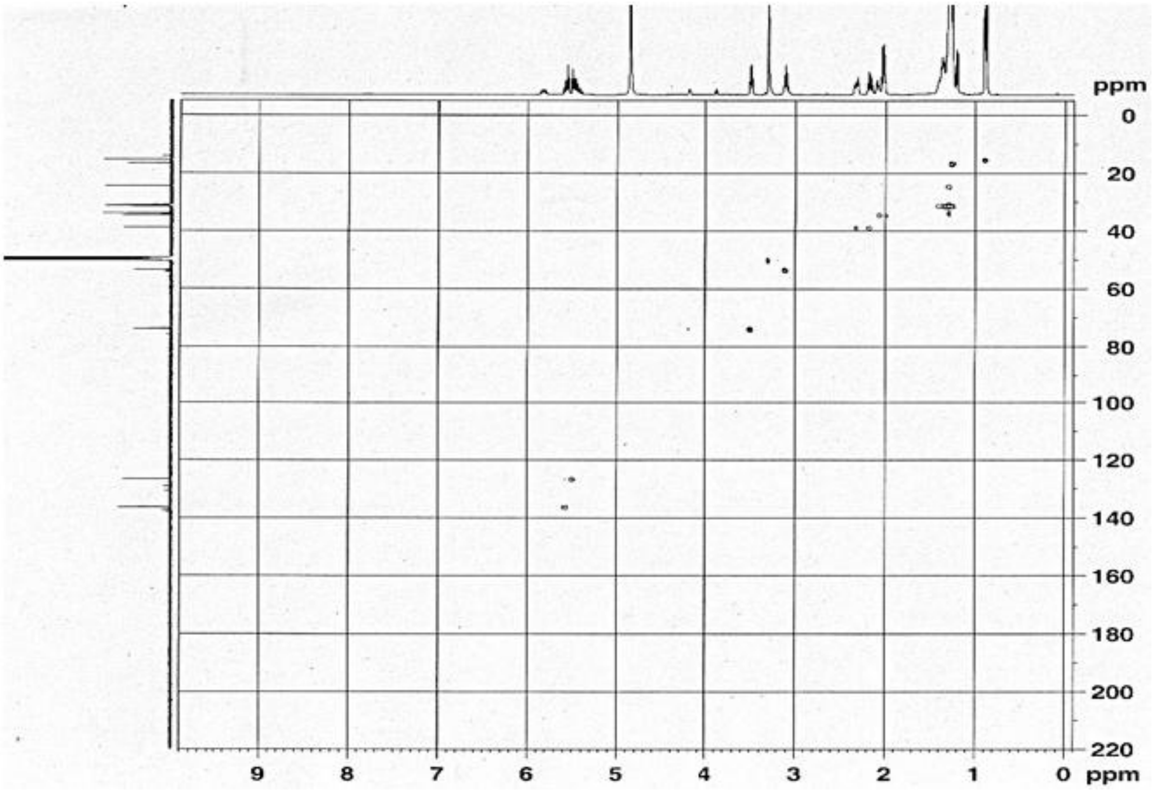

Figure S12. The *g*HMBC (600 MHz, MeOH-d_4) spectrum of pseudoaminol B (**2**).

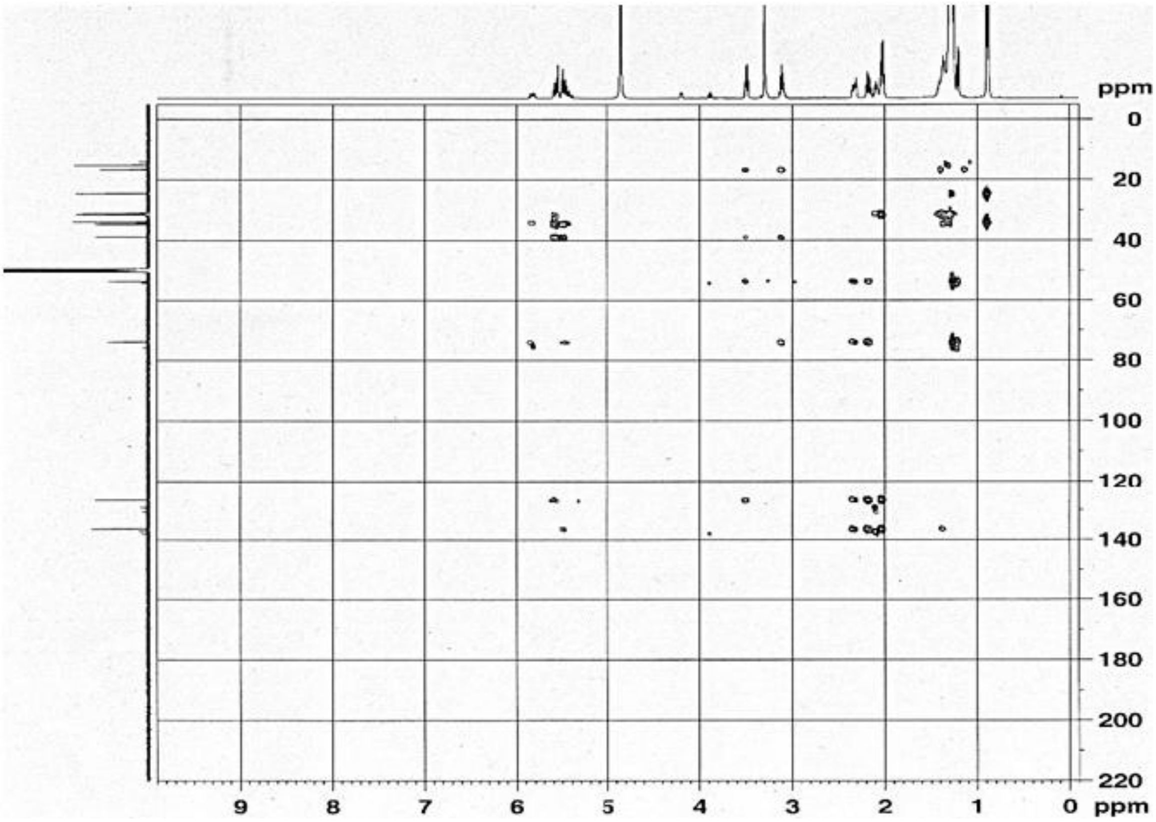

Figure S13. The ^{1}H NMR (600 MHz, MeOH-d_4) spectrum of pseudoaminol C (**3**).

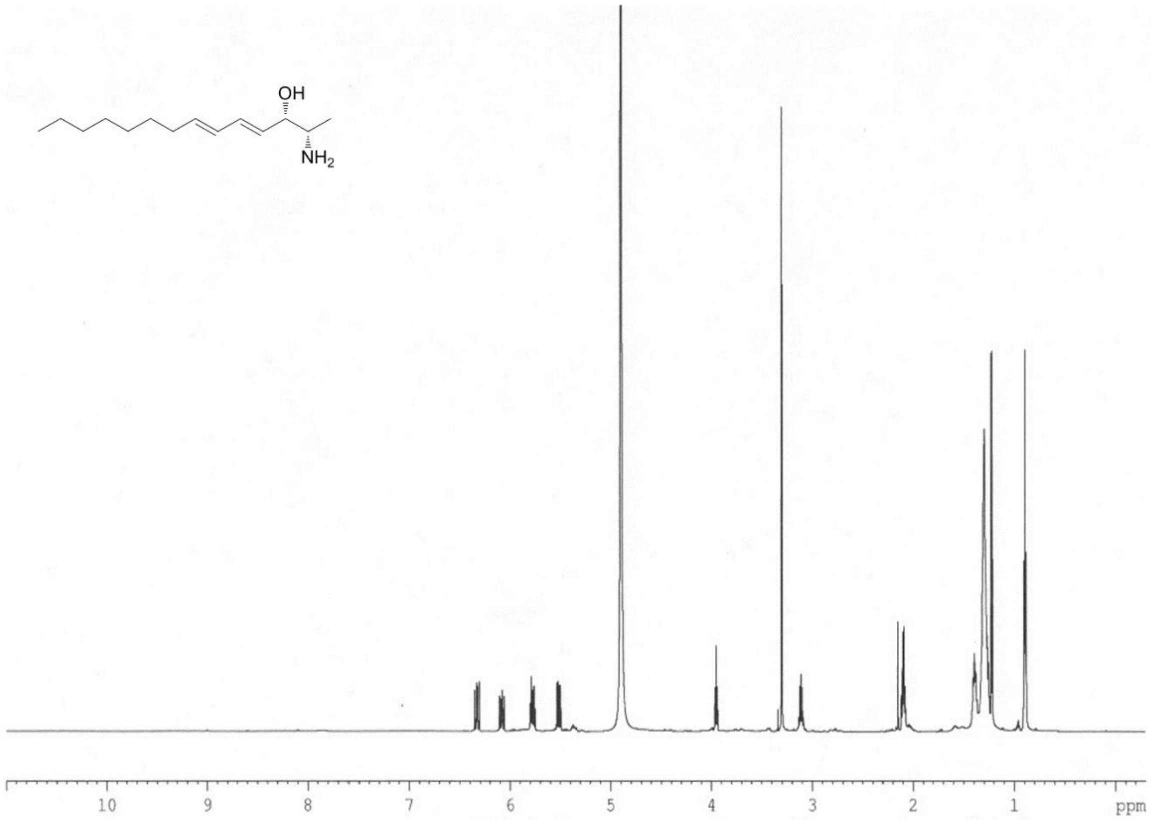

Figure S14. The ^{13}C NMR (150 MHz, MeOH-d_4) spectrum of pseudoaminol C (**3**).

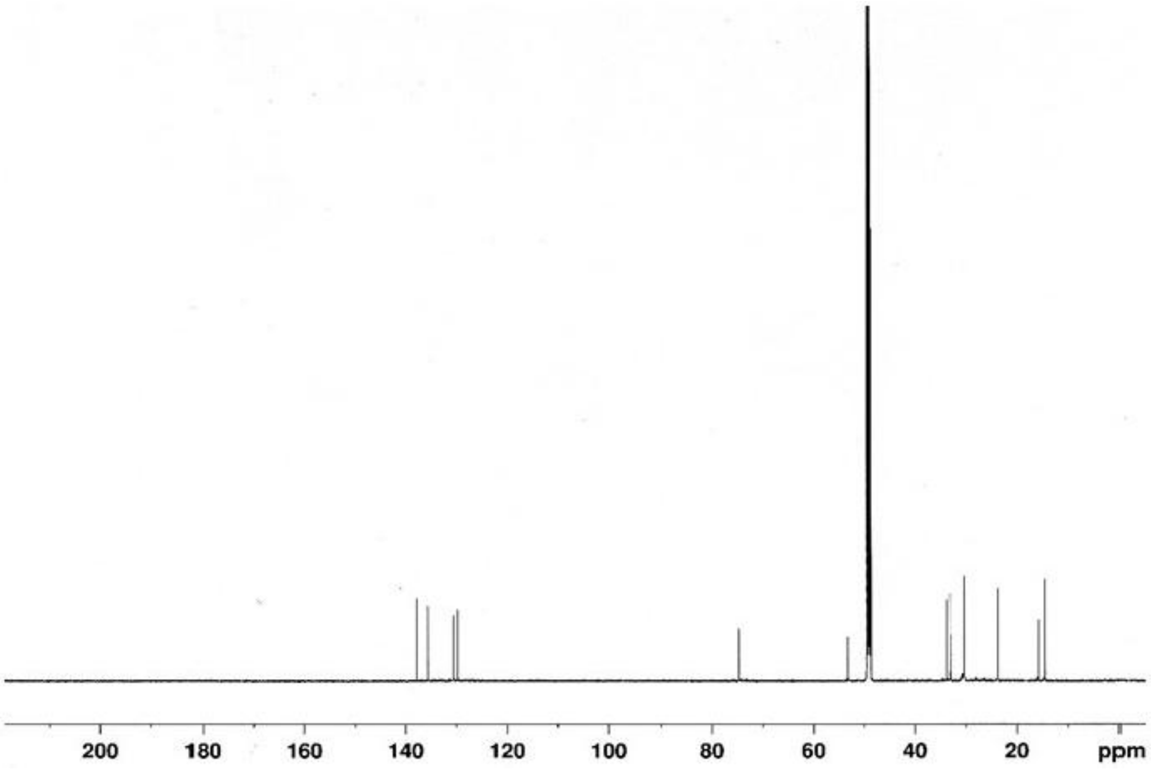

Figure S15. The COSY (500 MHz, MeOH-d_4) spectrum of pseudoaminol C (**3**).

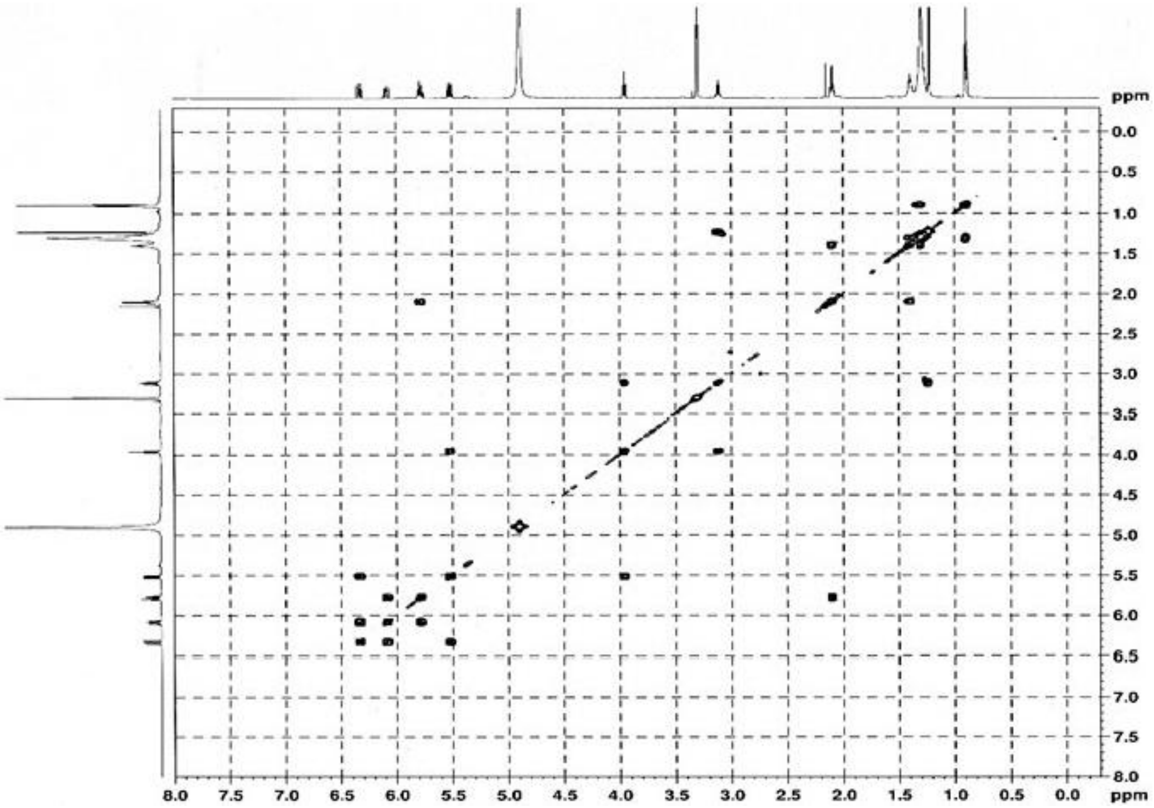

Figure S16. The *g*HSQC (500 MHz, MeOH-d_4) spectrum of pseudoaminol C (**3**).

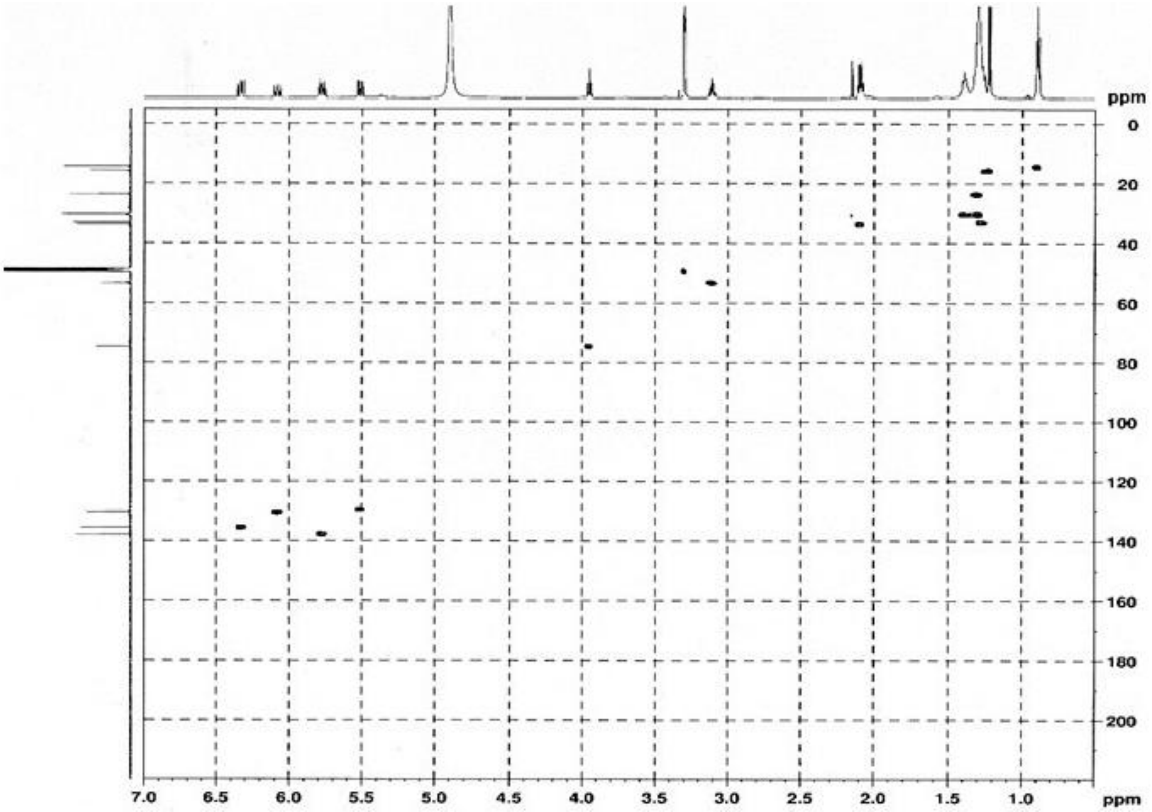

Figure S17. The *g*HMBC (500 MHz, MeOH-d_4) spectrum of pseudoaminol C (**3**).

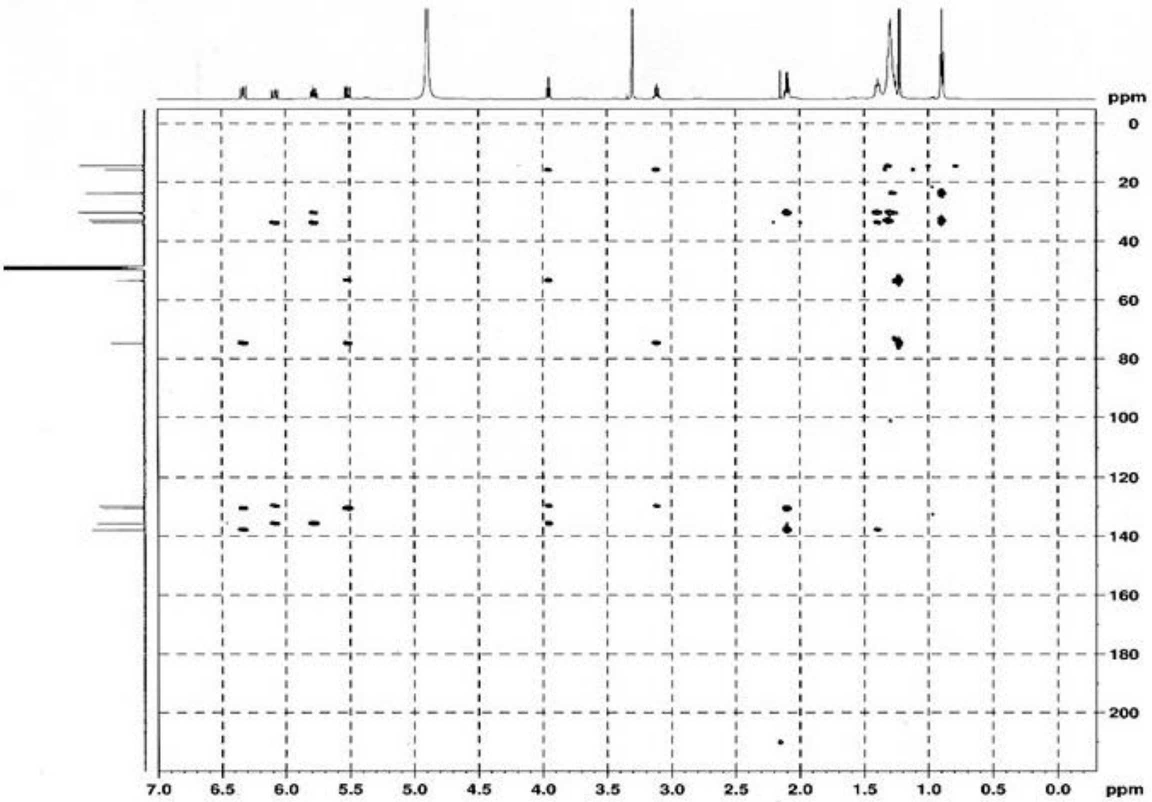

Figure S18. The ^{1}H NMR (600 MHz, MeOH-d_4) spectrum of pseudoaminol D (**4**).

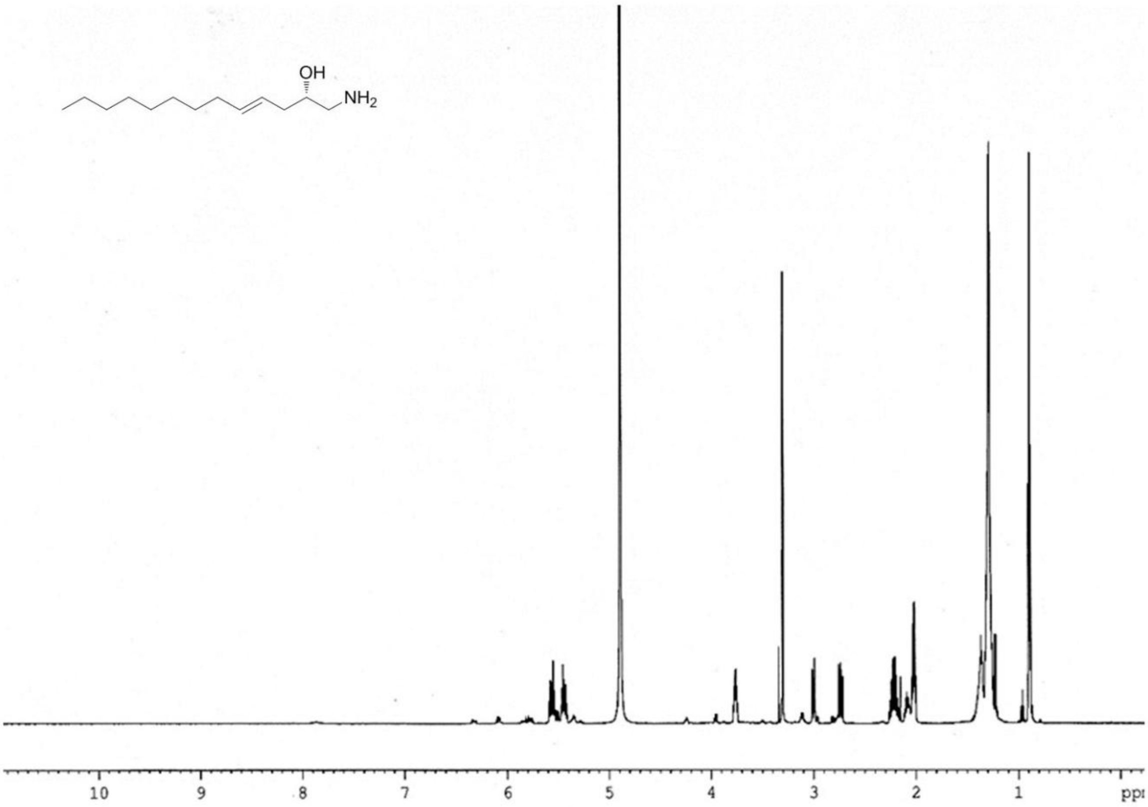

Figure S19. The ^{13}C NMR (150 MHz, MeOH-d_4) spectrum of pseudoaminol D (**4**).

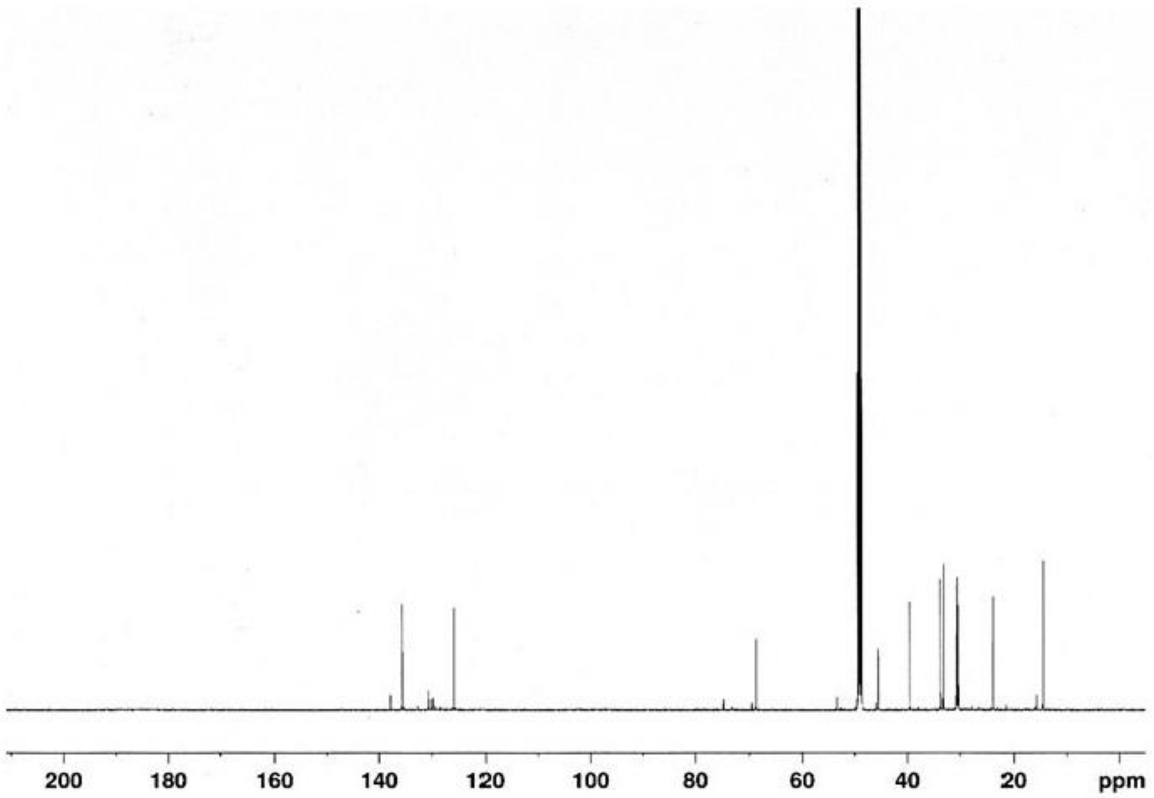

Figure S20. The COSY (600 MHz, MeOH-d_4) spectrum of pseudoaminol D (**4**).

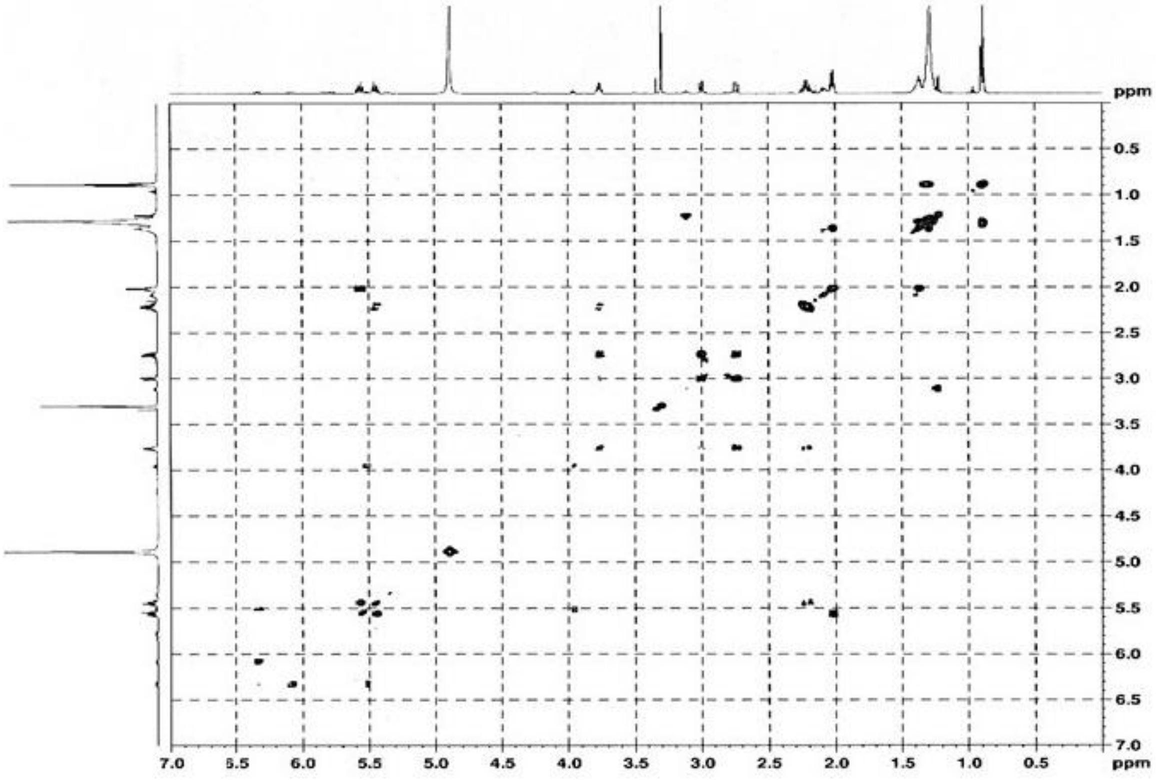

Figure S21. The *g*HSQC (600 MHz, MeOH-d_4) spectrum of pseudoaminol D (**4**).

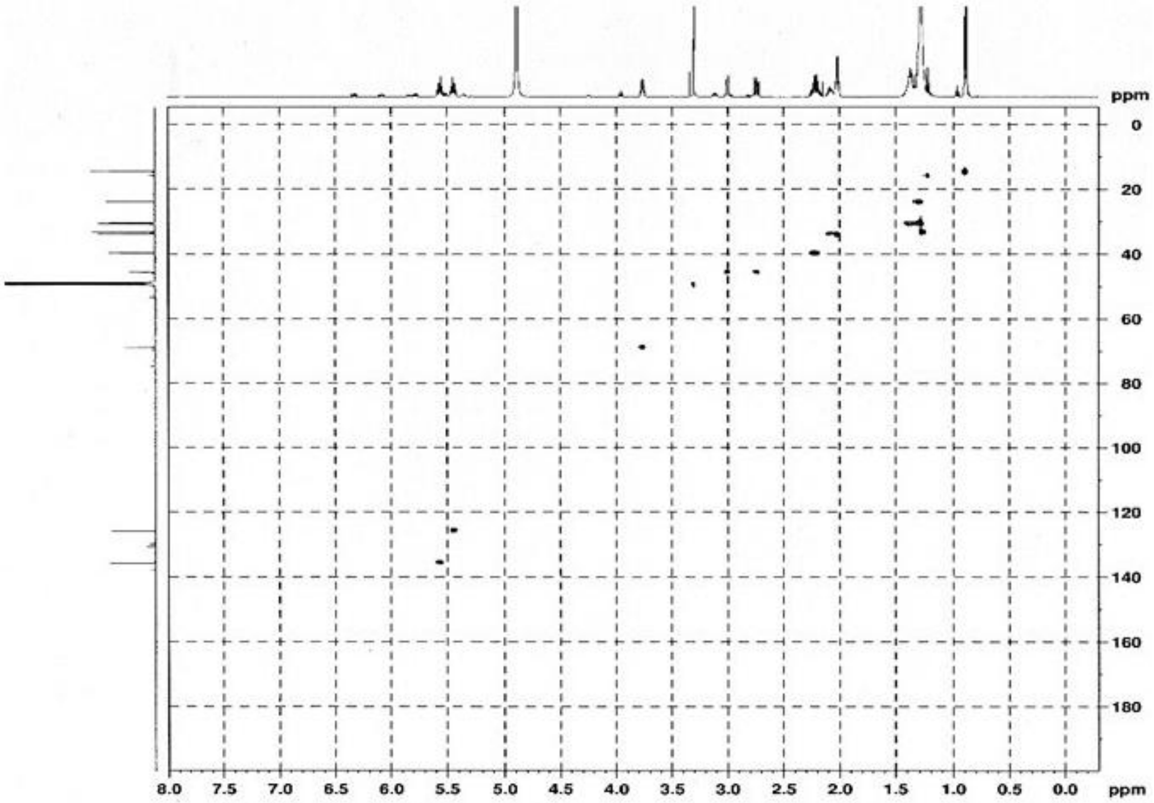

Figure S22. The *g*HMBC (600 MHz, MeOH-d_4) spectrum of pseudoaminol D (**4**).

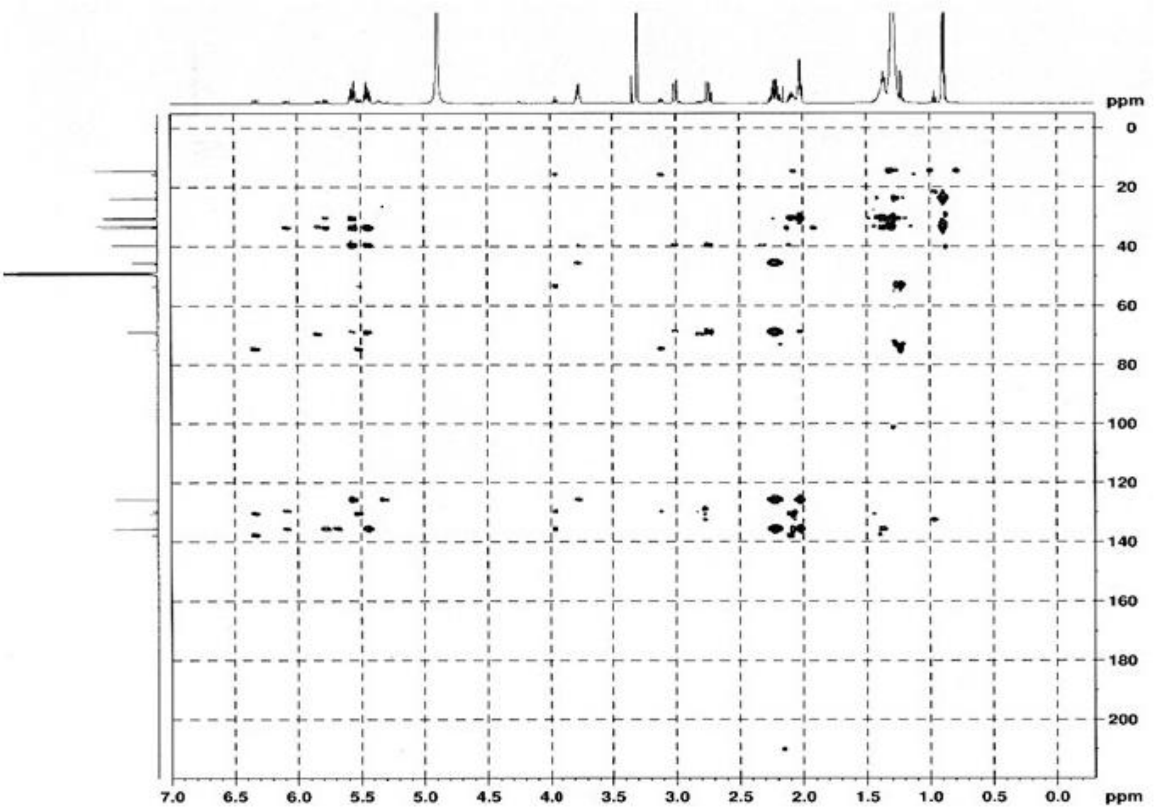

Figure S23. The ^{1}H NMR (500 MHz, MeOH-d_4) spectrum of pseudoaminol E (**5**).

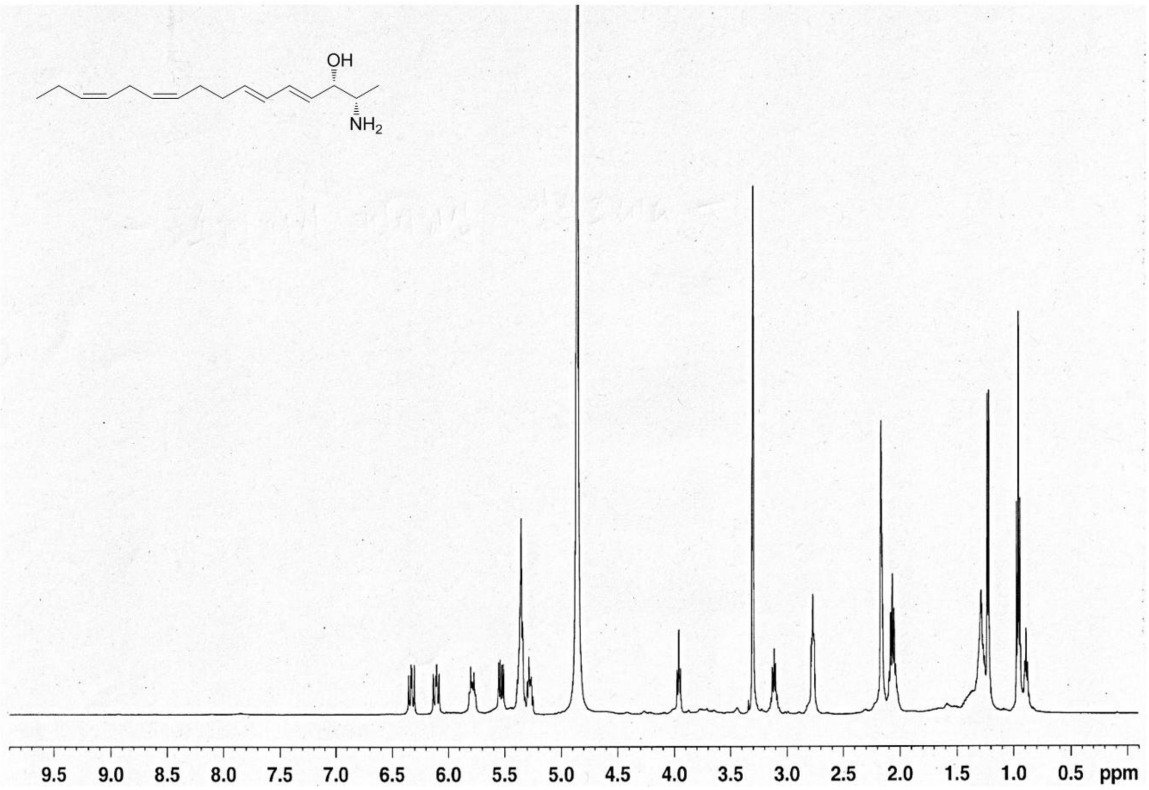

Figure S24. The ^{13}C NMR (125 MHz, MeOH-d_4) spectrum of pseudoaminol E (**5**).

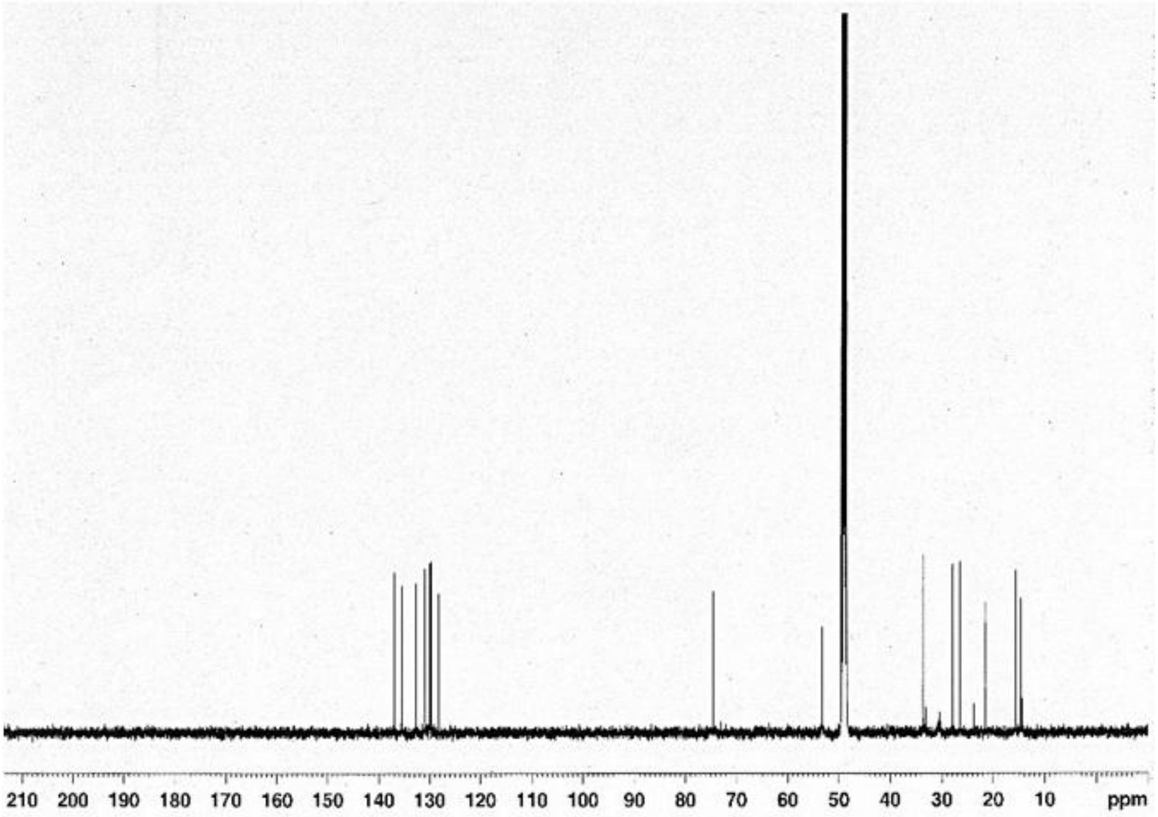

Figure S25. The COSY (500 MHz, MeOH-d_4) spectrum of pseudoaminol E (**5**).

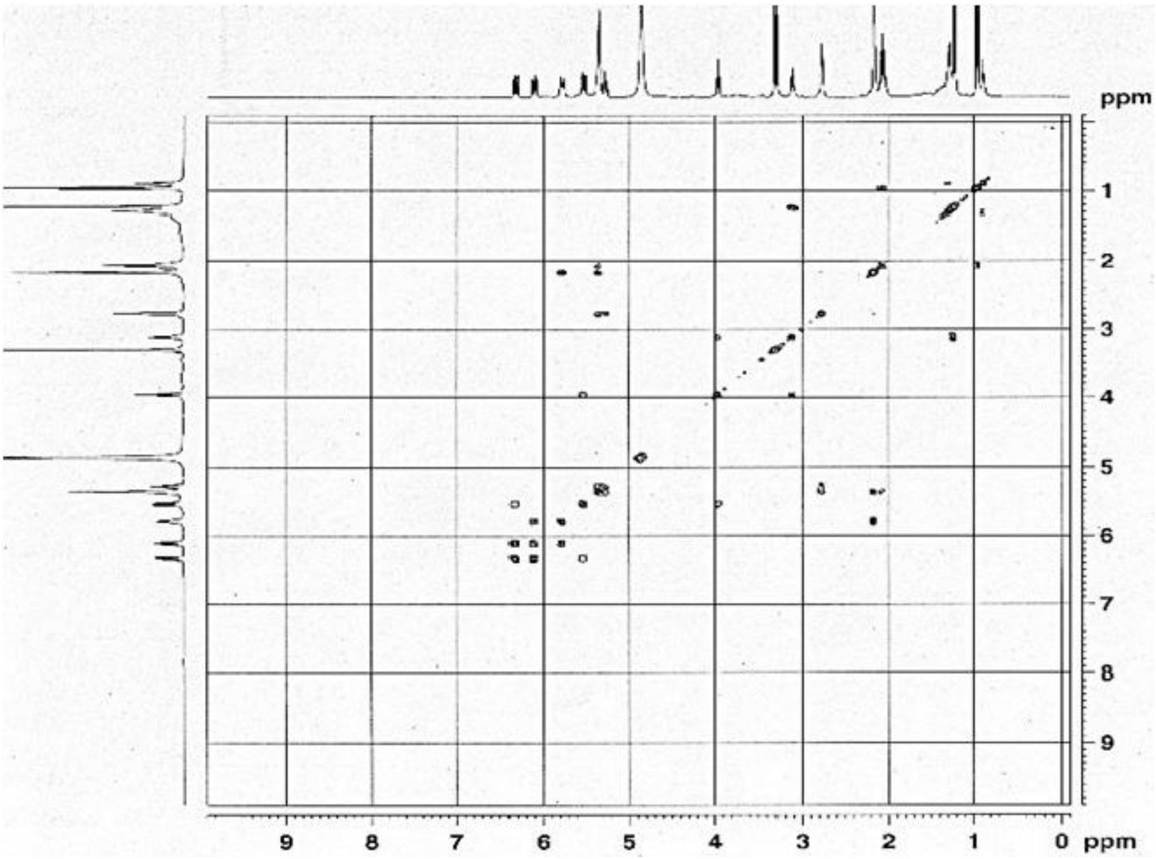

Figure S26. The *g*HSQC (500 MHz, MeOH-d_4) spectrum of pseudoaminol E (**5**).

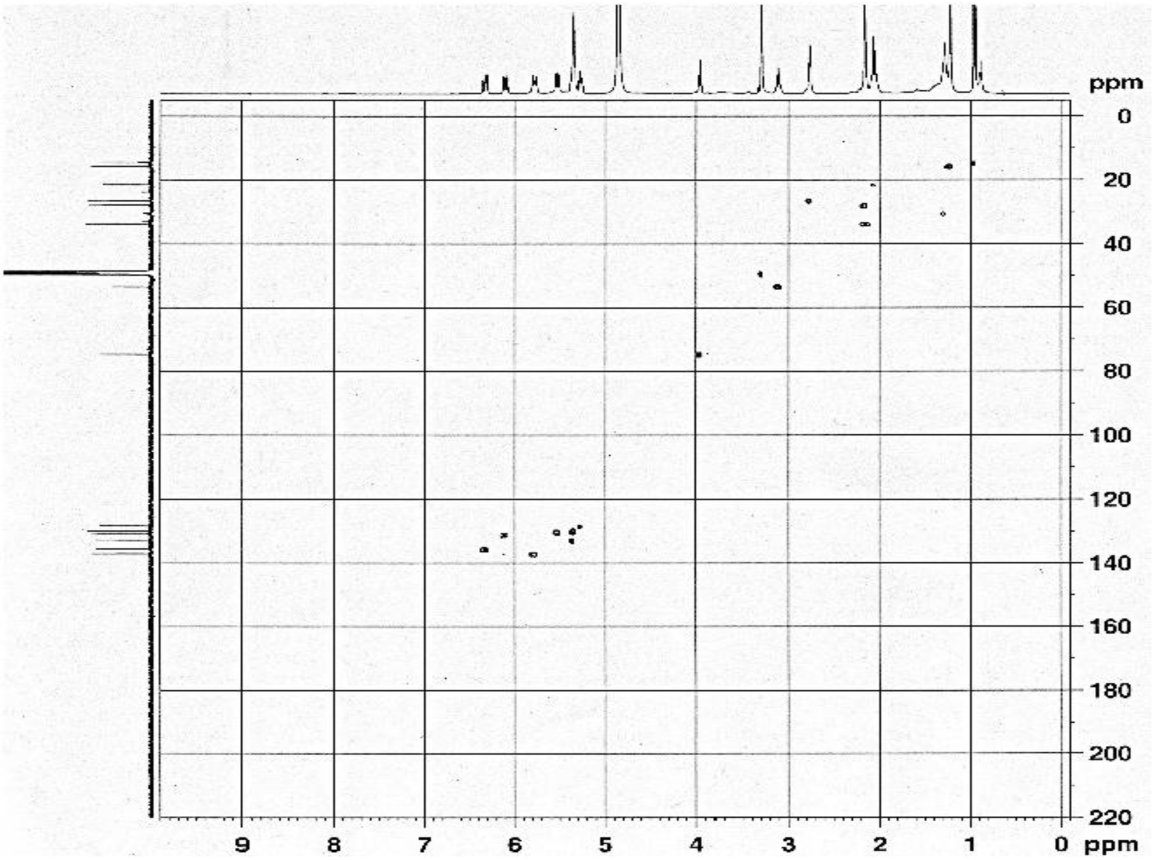

Figure S27. The *g*HMBC (500 MHz, MeOH-d_4) spectrum of pseudoaminol E (**5**).

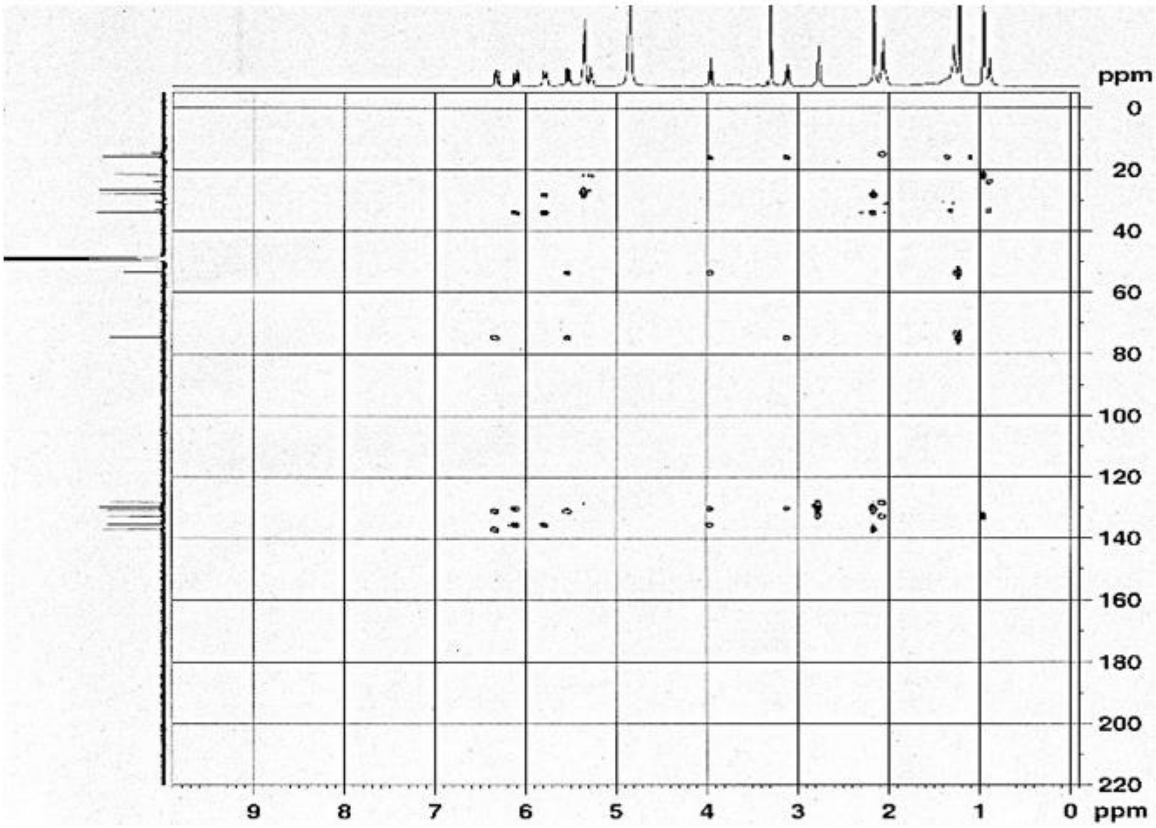

Figure S28. The ^{1}H NMR (600 MHz, DMSO-d_6) spectrum of pseudoaminol F (**6**).

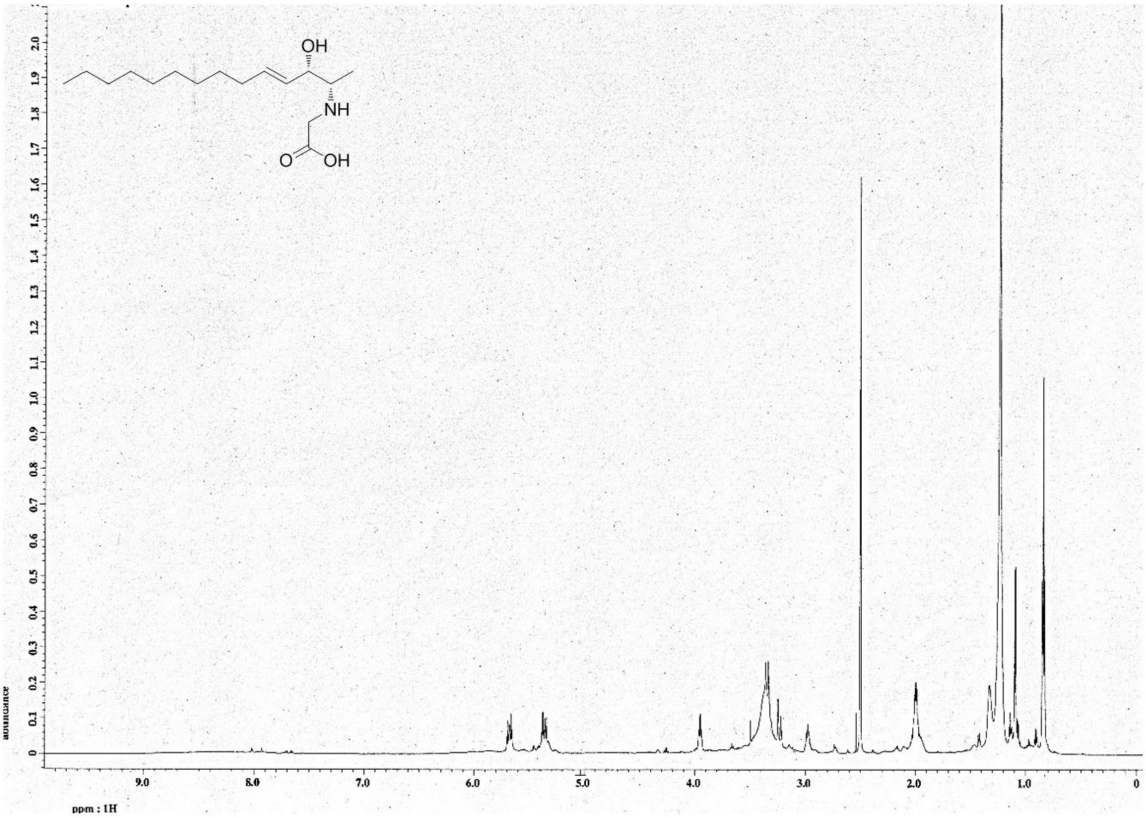

Figure S29. The ^{13}C NMR (150 MHz, DMSO-d_6) spectrum of pseudoaminol F (**6**).

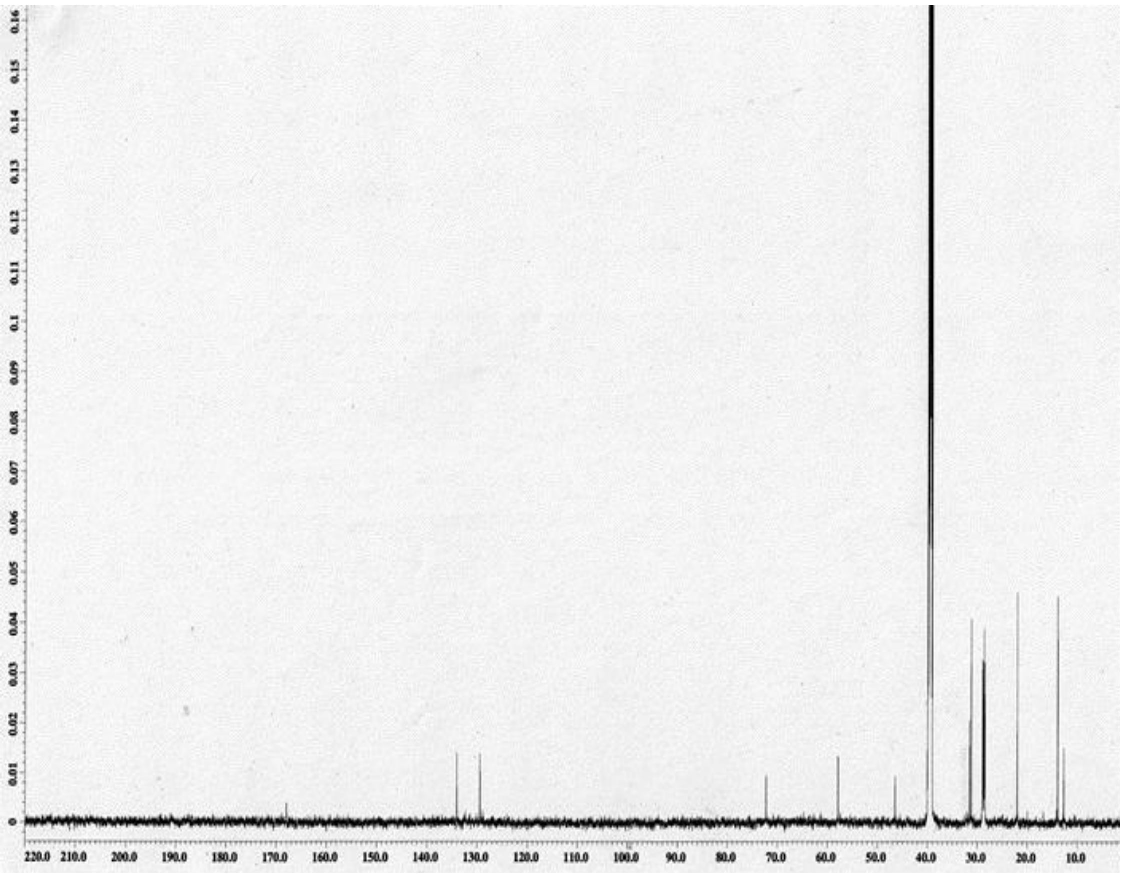

Figure S30. The COSY (600 MHz, DMSO-d_6) spectrum of pseudoaminol F (**6**).

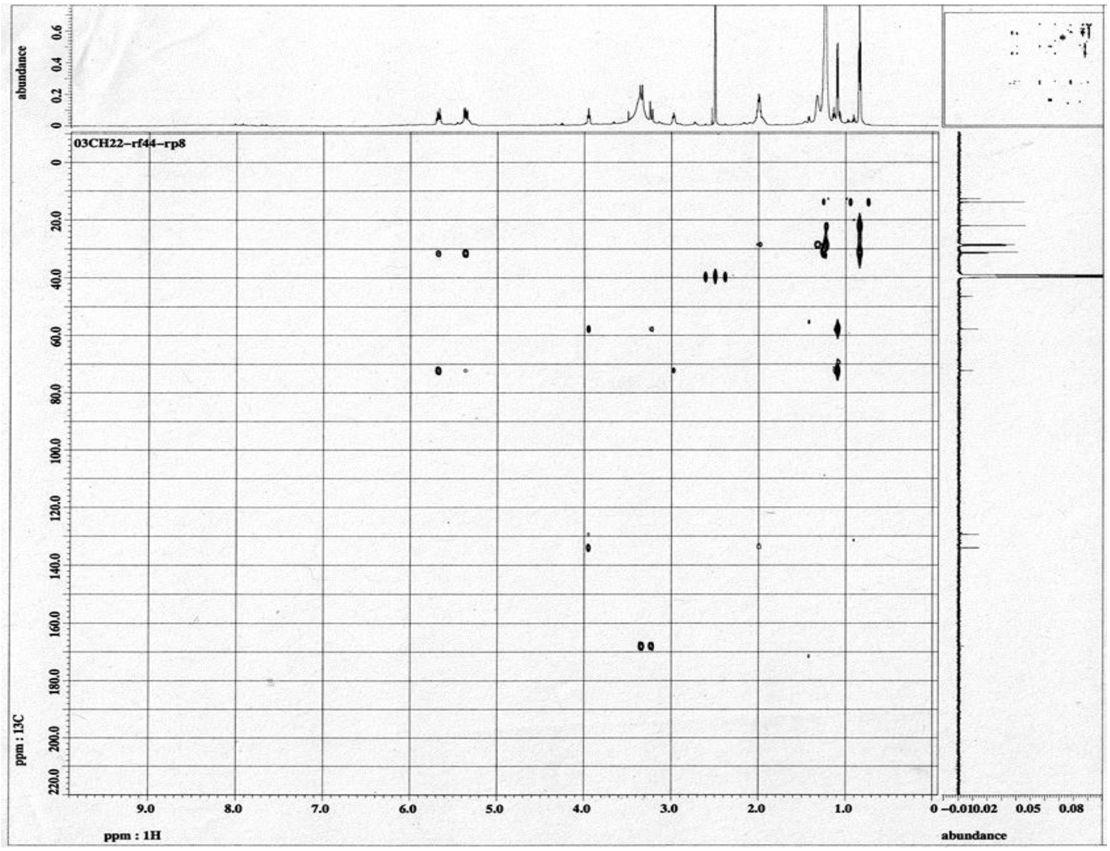

Figure S31. The *g*HSQC (600 MHz, DMSO-d_6) spectrum of pseudoaminol F (**6**).

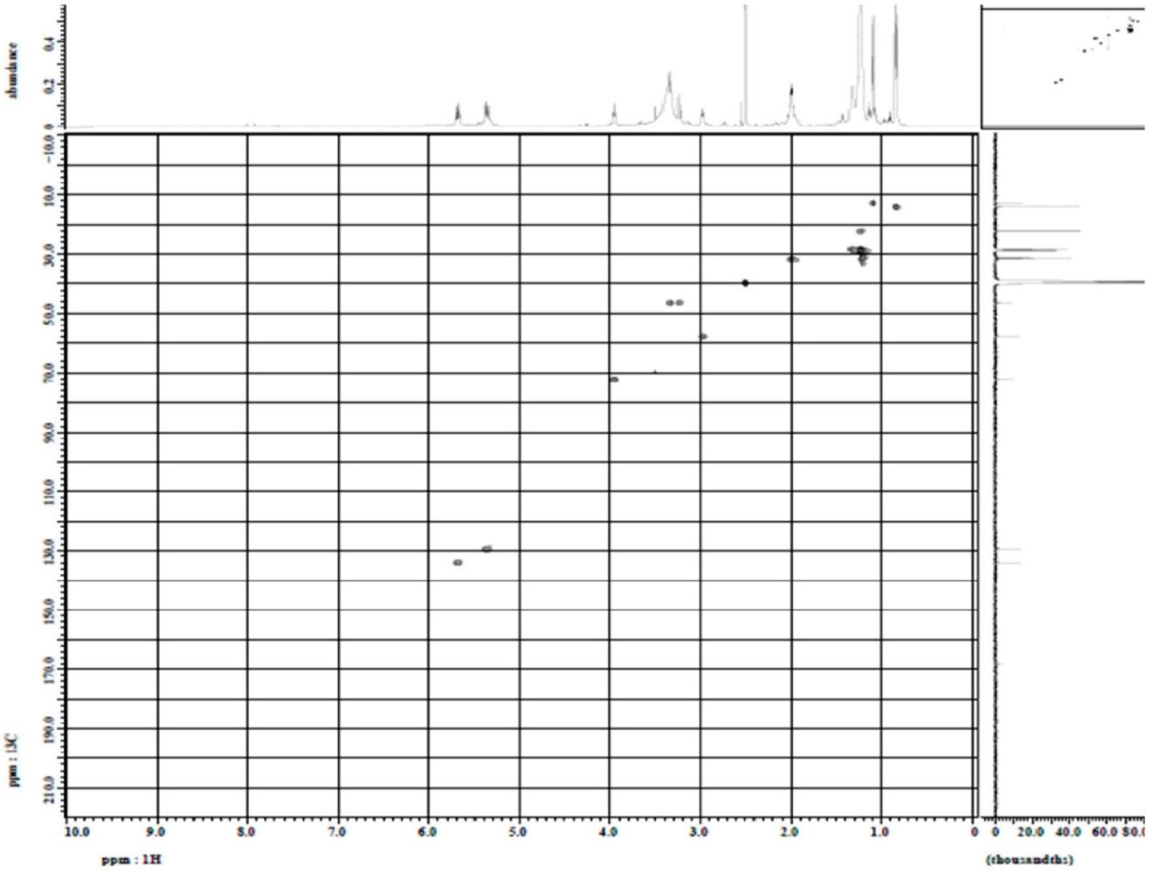

Figure S32. The *g*HMBC (600 MHz, DMSO-d_6) spectrum of pseudoaminol F (**6**).

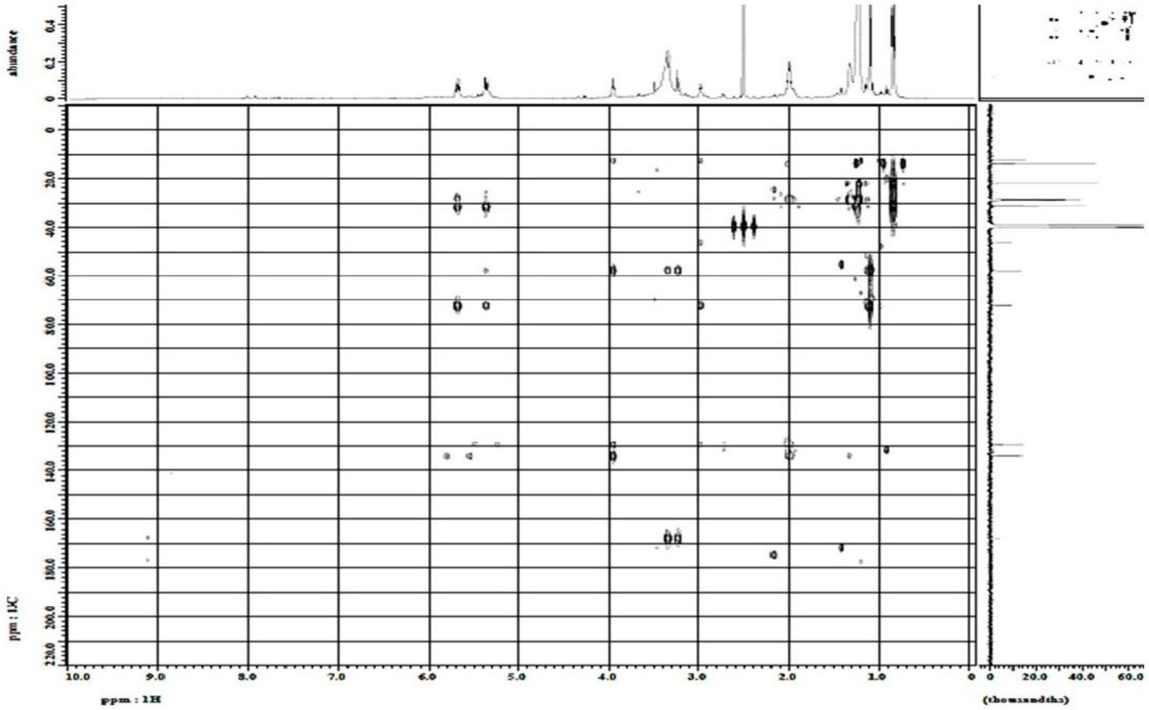

Figure S33. The ^{1}H NMR (600 MHz, DMSO-d_6) spectrum of pseudoaminol G (**7**).

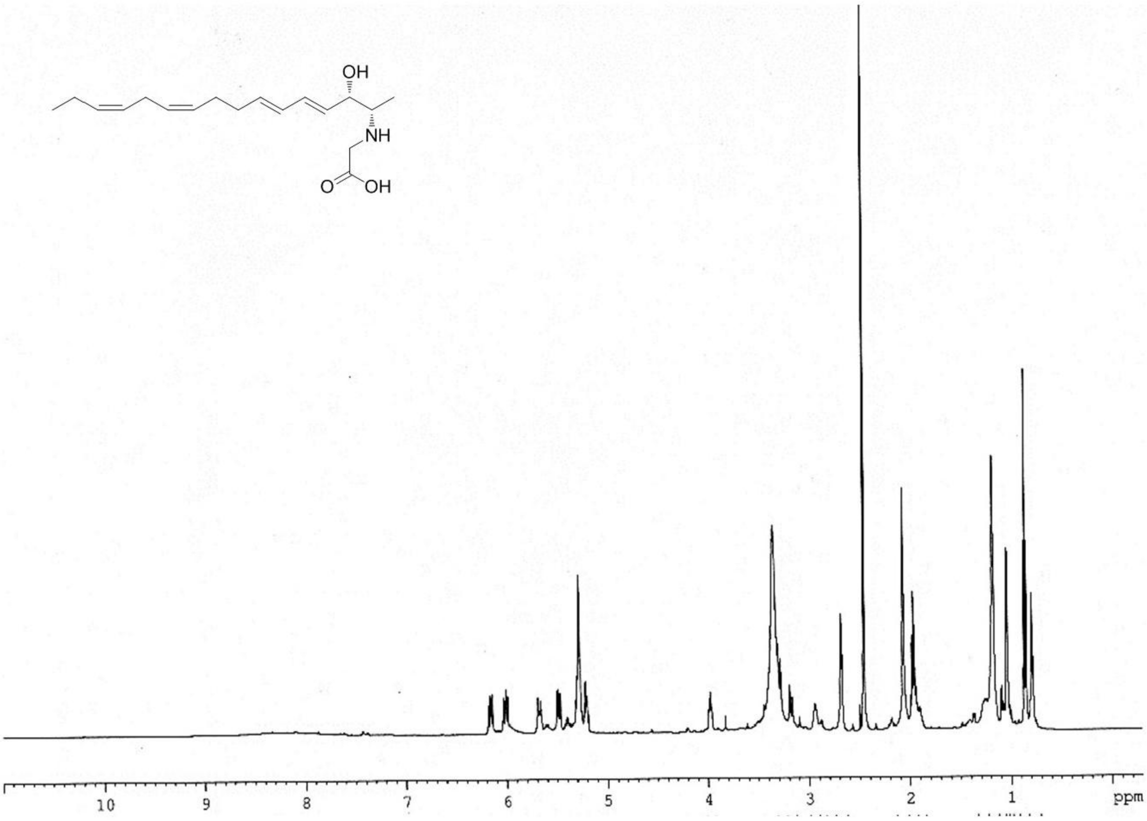

Figure S34. The ^{13}C NMR (150 MHz, DMSO-d_6) spectrum of pseudoaminol G (**7**).

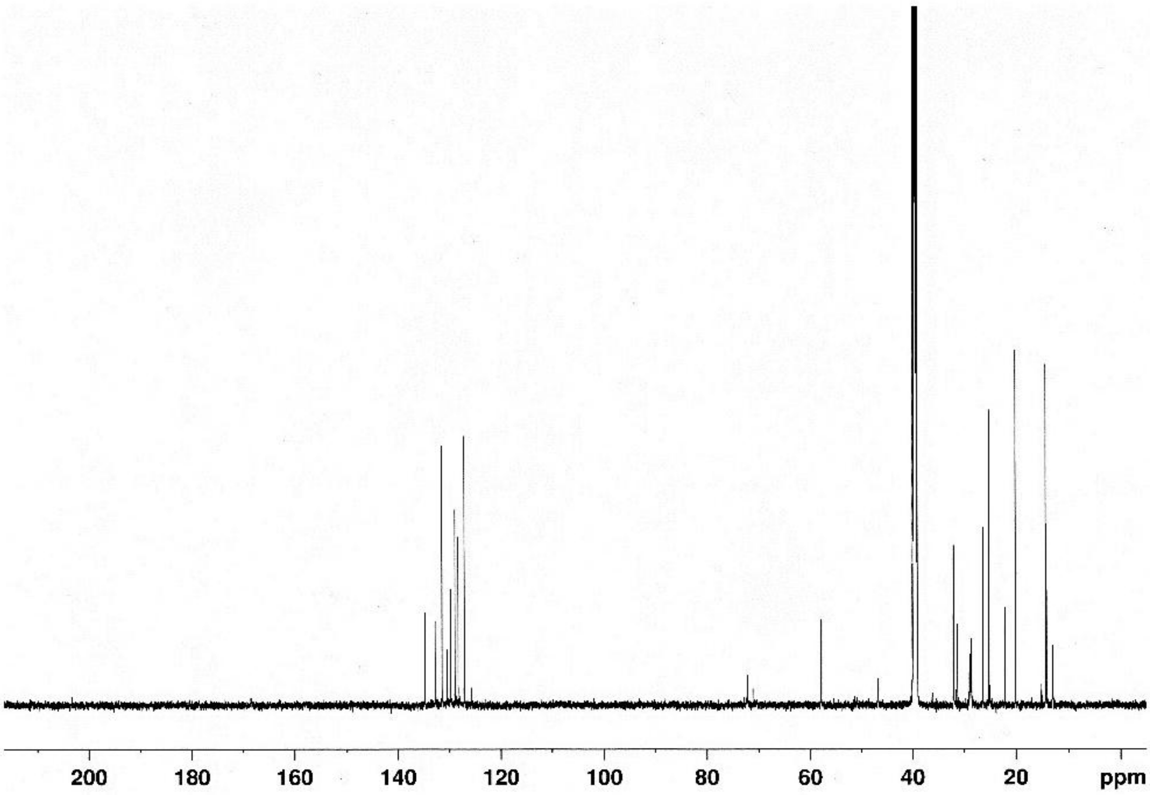

Figure S35. The COSY (600 MHz, DMSO-d_6) spectrum of pseudoaminol G (**7**).

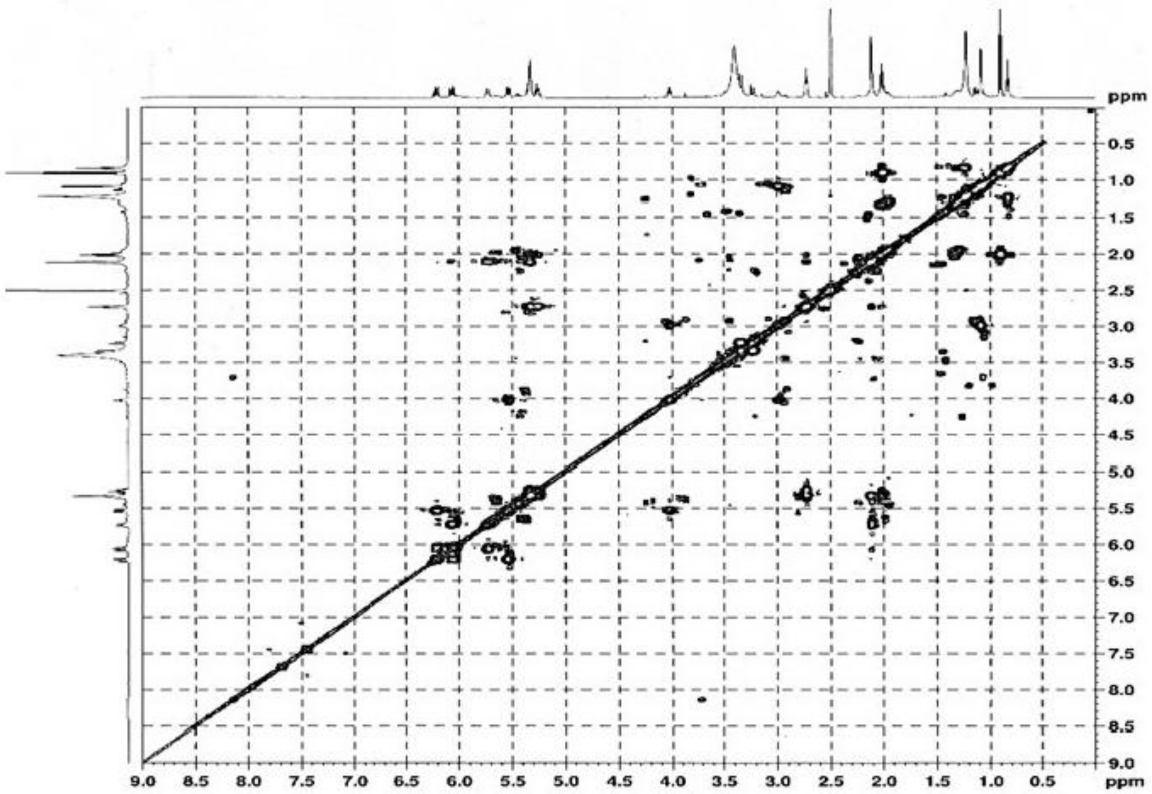

Figure S36. The *g*HSQC (600 MHz, DMSO-d_6) spectrum of pseudoaminol G (**7**).

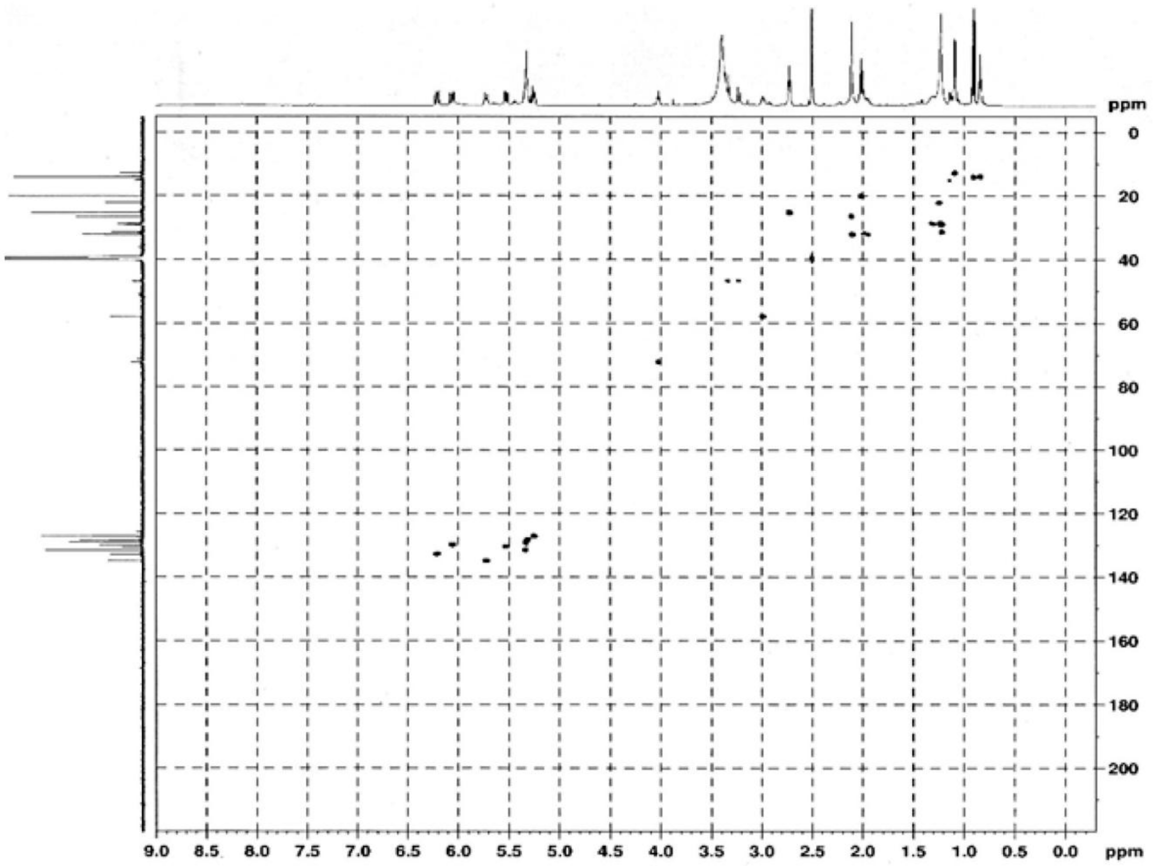

Figure S37. The *g*HMBC (600 MHz, DMSO-d_6) spectrum of pseudoaminol G (**7**).

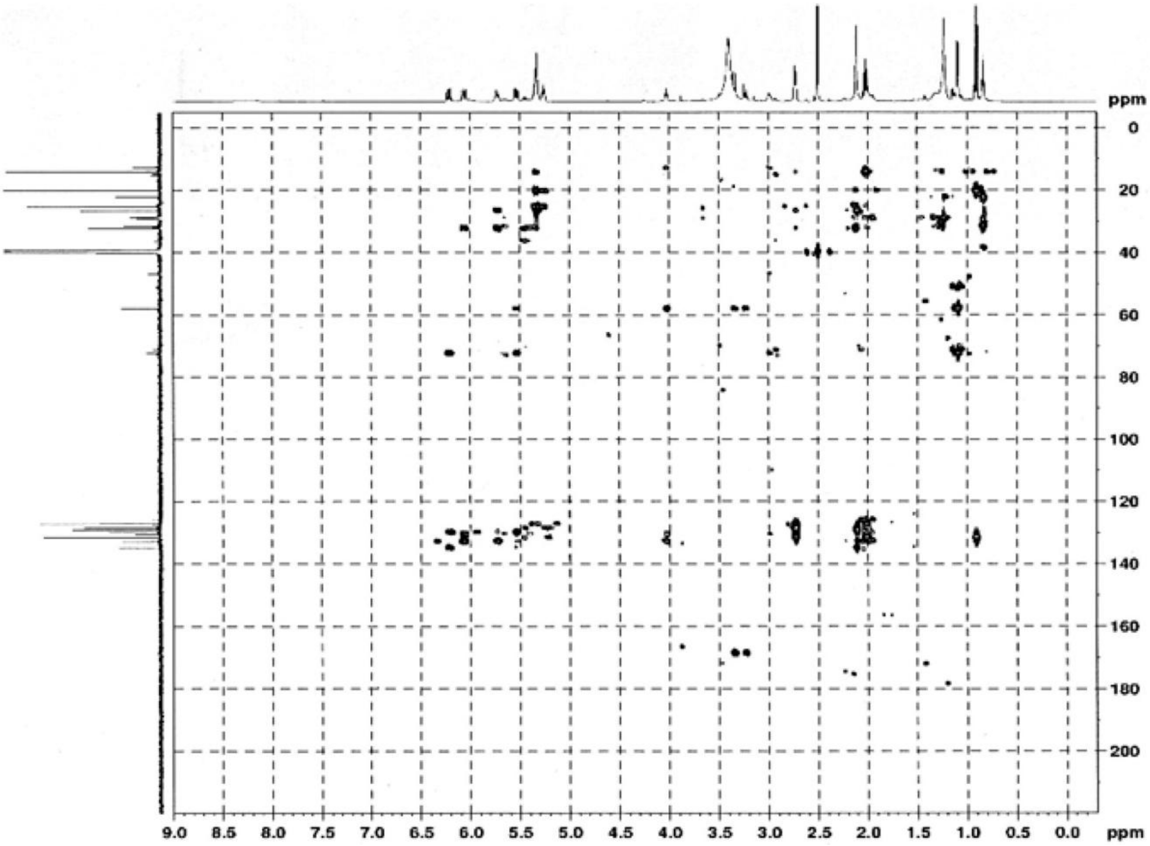

© 2014 by the authors; licensee MDPI, Basel, Switzerland. This article is an open access article distributed under the terms and conditions of the Creative Commons Attribution license (http://creativecommons.org/licenses/by/3.0/).
